# CD63 as novel target for nanoemulsion-based ^19^F MRI imaging and drug delivery to activated cardiac fibroblasts

**DOI:** 10.7150/thno.96990

**Published:** 2025-01-01

**Authors:** Arlen Aurora Euan Martínez, Ann Kathrin Bergmann, Frederik Tellkamp, Stephan Schott-Verdugo, Pascal Bouvain, Julia Steinhausen, Jasmin Bahr, Vivien Kmietczyk, Maja Bencun, Ulrich Flögel, Jörg H. W. Distler, Marcus Krueger, Mirko Völkers, Constantin Czekelius, Holger Gohlke, Sebastian Temme, Julia Hesse, Jürgen Schrader

**Affiliations:** 1 Department of Molecular Cardiology, Medical Faculty and University Hospital Düsseldorf, Heinrich Heine University Düsseldorf, Düsseldorf, Germany.; 2 Crozet-Medical GmbH, Düsseldorf, Germany.; 3 Core Facility for Electron Microscopy, Medical Faculty and University Hospital Düsseldorf, Heinrich Heine University Düsseldorf, Düsseldorf, Germany.; 4 Institute for Genetics, University of Cologne, Cologne, Germany.; 5 Cologne Excellence Cluster on Stress Responses in Aging-associated Diseases (CECAD), University of Cologne, Cologne, Germany.; 6 Institute of Bio- and Geosciences (IBG-4: Bioinformatics), Forschungszentrum Jülich, Jülich, Germany.; 7 Department of Internal Medicine III (Cardiology, Angiology, and Pneumology), Heidelberg University Hospital, Heidelberg, Germany.; 8 DZHK (German Centre for Cardiovascular Research), partner site Heidelberg/Mannheim, Heidelberg, Germany.; 9 CARID, Cardiovascular Research Institute Düsseldorf, Medical Faculty and University Hospital Düsseldorf, Heinrich Heine University Düsseldorf, Düsseldorf, Germany.; 10 Department of Rheumatology, Medical Faculty and University Hospital Düsseldorf, Heinrich Heine University Düsseldorf, Düsseldorf, Germany.; 11 Hiller Research Center, Medical Faculty and University Hospital Düsseldorf, Heinrich Heine University Düsseldorf, Düsseldorf, Germany.; 12 Institute for Organic Chemistry and Macromolecular Chemistry, Heinrich Heine University Düsseldorf, Düsseldorf, Germany.; 13 Institute for Pharmaceutical and Medicinal Chemistry, Heinrich Heine University Düsseldorf, Düsseldorf, Germany.; 14 Department of Anesthesiology, Medical Faculty and University Hospital Düsseldorf, Heinrich Heine University Düsseldorf, Düsseldorf, Germany.

**Keywords:** myocardial infarction, fibrosis, CD63, FAP, modified mRNA

## Abstract

**Rationale:** Cardiac fibroblasts are activated following myocardial infarction (MI) and cardiac fibrosis is a major driver of the growing burden of heart failure. A non-invasive targeting method for activated cardiac fibroblasts would be advantageous because of their importance for imaging and therapy.

**Methods:** Targeting was achieved by linking a 7-amino acid peptide (EP9) to a perfluorocarbon-containing nanoemulsion (PFC-NE) for visualization by ^19^F-combined with ^1^H-MRI. *In vivo* and *ex vivo* ^1^H/^19^F MRI was performed on a Bruker 9.4 T AVANCE III wide-bore nuclear magnetic resonance spectrometer. Photoaffinity labeling (diazirine photolinker) and mass spectrometry were used to identify the peptide-binding protein. Molecular modeling studies used ColabFold and AlphaFold 3. EP9-decorated liposomes containing modified mRNA for luciferase (mRNA-LUC) were used for the study of the cellular uptake process.

**Results:** After injection of EP9-PFC-NE, the in-vivo ^19^F signal localized to the infarcted area of the heart and was EP9-specific, as verified by the use of a mutated peptide. The plasma half-life of the nanoemulsion was 20 h and electron microscopy identified cardiac fibroblasts and epicardial stromal cells to be the main populations for cellular uptake. Photoaffinity labeling identified the tetraspanin CD63 as the main EP9-binding protein, which was supported by CD63-EP9 modeling data. Expression of CD63 was significantly upregulated in infarct-activated fibroblasts of mice and humans. Cellular uptake may involve caveolae and/or clathrin-coated pits as suggested by scRNAseq data. Uptake studies with mRNA-LUC-loaded EP9-PFC-NE confirmed internalization after binding to fibroblast CD63.

**Conclusions:** CD63 was identified to contain a specific EP9 binding motive that triggers endocytosis of EP9-PFC-NE in activated cardiac fibroblasts. This targeted nanoemulsion can therefore be used for *in vivo* imaging and has the potential for fibroblast-specific drug delivery.

## Introduction

After myocardial infarction (MI), the heart tissue undergoes a complex process of healing and remodeling, and fibroblasts play a crucial role in this process 1. They are responsible for the synthesis and deposition of collagen forming the extracellular matrix that provides structural support, but over time also can lead to impaired cardiac function and finally heart failure. Fibroblasts also facilitate the healing process by promoting the formation of new blood vessels. In addition, they modulate the inflammatory response that occurs after MI, which may result in a prolonged inflammation that contributes to pathological remodeling 1. Overall, fibroblasts are integral to the repair and remodeling of the heart following MI, and understanding their role and mechanisms of action can potentially lead to the development of novel diagnostic and therapeutic strategies.

During development cardiac fibroblasts are formed from various cell lineages. Epicardium-derived stromal cells (EpiSC), originating from the epicardium by epithelial-to-mesenchymal transition (EMT), are considered to be a main contributor to cardiac fibroblasts 2. In the adult heart, the epicardium is a rather quiescent monolayer. After MI, epicardial cells covering the injured myocardium become activated 3 and generate a multi-cell layer of EpiSC in the subepicardium that can reach a thickness of about 50-70 µm in mice 4,5. Adult EpiSC secrete paracrine factors that stimulate cardiomyocyte growth and angiogenesis 6, play a key role in post-MI adaptive immune regulation 7, and even can form cardiomyocytes 8. Thus, the epicardium similar to cardiac fibroblasts is a signaling center regulating cardiac wound healing in the adult injured heart.

Noninvasive methods for targeting individual cell types of the heart in a specific manner are of growing importance for diagnosis and future therapy. Magnetic resonance imaging (^1^H MRI) is well-established for cardiac visualization with high spatial resolution and high contrast. Combined with fluorine (^19^F)-mediated labeling, ^1^H/^19^F MRI has the advantage that ^19^F-labeled cells can be tracked *in vivo* with high sensitivity since ^19^F MRI is essentially background-free 9. ^19^F-based perfluorocarbon-containing nanoemulsions (PFC-NE) coupled for specific targeting to ligands by the sterol-based postinsertion technique have been demonstrated by us as a unique platform for early thrombus detection 10 and mapping of neutrophil dynamics 11 by *in vivo* ^19^F-MRI. This hot spot imaging technique has recently been used to characterize a wide array of cardiovascular diseases 12. This imaging modality can complement other imaging techniques such as gadolinium for scar assessment and manganese, a calcium analogue, that is useful for imaging myocardial viability.

An additional modality for cell-specific imaging in the heart is positron emission computed tomography (PET). The membrane-bound serine protease FAP (fibroblast activation protein) was recently successfully used to visualize infarct-activated cardiac fibroblasts via PET in patients with a history of coronary artery disease 13. Successful application of this technique has also been reported for cardiac fibroblasts in pulmonary arterial hypertension 14, dilated cardiomyopathy 15, and after chemotherapy or chest radiotherapy 16. Whether FAP can also be used for targeted delivery of e.g. therapeutic substances to activated cardiac fibroblasts has not been explored yet.

Using bacterial phage display screening, our previous work identified five small peptides that bound to cultured EpiSC, isolated from infarcted rat hearts, with high affinity 17. One peptide coined EP9 (Ac-KLMLPRPGGGKC-NH_2_) showed the highest affinity and specificity among all tested peptides and therefore was explored in the present study as a candidate peptide to target PFC-NE to cardiac stromal cells in an MI mouse model. Given the developmental relationship between EpiSC and cardiac fibroblasts, we explored whether EP9-coupled PFC-NE (EP9-PFC-NE) bind to infarct-activated cardiac fibroblasts and can be used for MRI. We used photoaffinity labeling to identify cell surface proteins to which EP9 preferentially binds and performed first proof-of-principle experiments to evaluate EP9-decorated liposomes as drug delivery system.

## Materials and methods

### Animal experiments

Animal protocols were approved by the North Rhine-Westphalia Office of Nature, Environment and Consumer Protection (LANUV) (reference number: AZ 81‑02.04.2019.A466). For this study, male C57BL/6J mice (Janvier Labs, Le Genest-Saint-Isle, France) with body weight: 20-25 g and age: 8-12 weeks were used. Mice were housed at the Central Institution for Animal Research and Scientific Animal Welfare (ZETT) of the Heinrich Heine University Düsseldorf, Düsseldorf, Germany, on a 12 h light/dark cycle, were fed with a standard chow diet, and received tap water ad libitum. Ischemia/reperfusion was induced as previously described 18. In brief, mice were anesthetized (1.5% (v/v) isoflurane via respiration), and the left anterior descending coronary artery (LAD) was ligated for 50 min followed by reperfusion. LAD occlusion was ensured by ST-segment elevation in ECG recordings. Mice were kept under analgesic treatment for 3 days (0.1 mg/kg buprenorphine, three times/day).

### Synthesis of targeted peptides and control peptides

**Figure 1:**
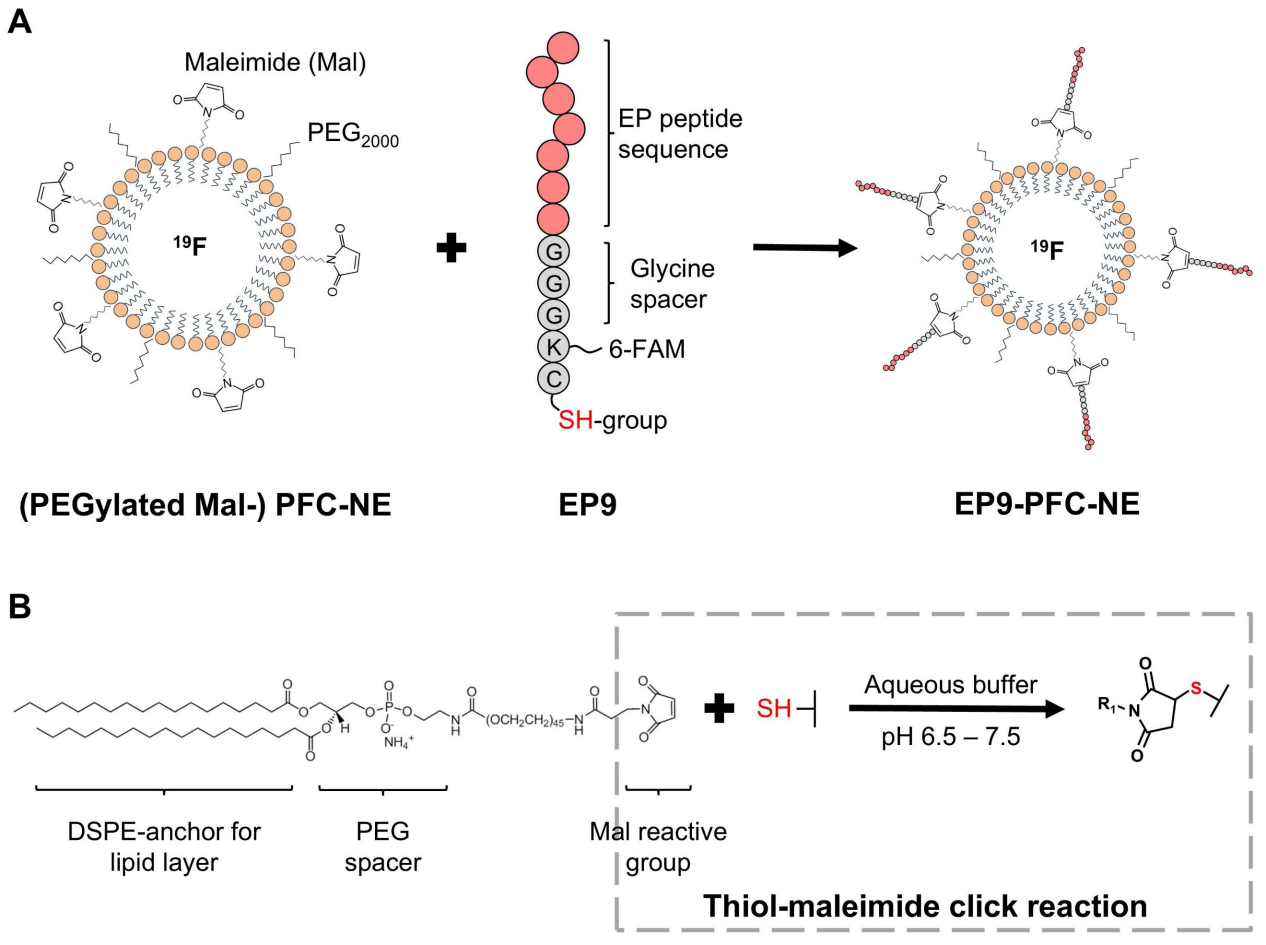
** Coupling of peptide EP9 to a perfluorocarbon-containing nanoemulsion (PFC-NE). A**: Scheme of preparing EP9-decorated PFC-NE (EP9-PFC-NE). Pre-manufactured nanoemulsions, composed of phospholipids that encapsulate perfluoro-15-crown-5 ether (PFC, with ^19^F), were PEGylated (PEG_2000_) and DSPE-PEG_2000_-maleimide (Mal) was attached at the surface. EP9, recently described by us 17, contains a cysteine amino acid displaying a reactive thiol (SH) group. **B:** In an aqueous buffer, the maleimide and thiol groups react and form a thioether bond, in this way covalently coupling EP9 to PFC‑NE.

Five small peptides (EP1, EP2, EP3, EP7, EP9) composed of 7 amino acids (Table S1) that specifically bind to EpiSC as described in our previous work 17 were synthesized commercially (Genaxxon Bioscience, Ulm, Germany). The fluorescent dye carboxyfluorescein (6-FAM) was coupled to the ε-amino group of the lysine attached to the glycine spacer (GGGK(FAM)C, see Figure 1A) to enable flow cytometry. The terminal cysteine was used for conjugation to the PFC‑NE. The EP9 sequence (Ac-KLMLPRPGGGKC-NH_2_) was used as a basis to design scrambled and mutated peptides. As depicted in Table S1, the amino acid positions were randomly interchanged and lysine was exchanged for glycine to obtain the scrambled peptide. The mutated peptide was obtained by replacing the proline residues with glycine and alanine.

### Preparation of the PFC-NE

PFC-NE were manufactured as described before 19 by microfluidization technology. In brief, phospholipid E80S (phosphatidylcholine content: approximately 70%, from soybean), perfluoro-15-crown-5 ether, and DSPE-PEG_2000_-maleimide (1,2-distearoyl-sn-glycero-3-phosphoethanolamine-N-[maleimide(polyethyleneglycol)-2000]) were magnetically stirred in 10 mM phosphate buffer (7 mM Na_2_HPO_4_, 3 mM NaH_2_PO_4_, pH 7.4 isotonized with 2.5% (w/w) glycerol) for 30 min at 300 rpm. Subsequently, the emulsion was formed using a homogenizer (Ultra-Turrax, IKA T18 basic; IKA Works, Wilmington, NC, USA) for 5 min. Afterwards, the emulsion was processed in a high-pressure homogenizer on a Low Volume Microfluidizer (Microfluidics, Westwood, MA, USA) for 9 cycles at 16,000 psi (≈1,000 bar). Nanoemulsions contained 20% w/w of perfluoro-15-crown-5 ether.

### Preparation of targeted PFC-NE

To generate PFC-NE for the *in vivo* targeting experiments, we performed a coupling to the peptide EP9 (KLMLPRP), which according to our previous work exhibited high binding activity to rat EpiSC 17, generating EP9-PFC-NE. As a control, we coupled PFC-NE to the mutated peptide (MUT, see Table S1), generating MUT-PFC-NE. The coupling reaction was performed as described before 17, using a thiol-maleimide click reaction between the thiol group of the cysteine at the N-terminus of the peptide (Figure 1A) and the maleimide anchored in the PFC-NE lipid layer (Figure 1B) forming a stable thioether bond. Briefly, 25 µg of the peptide were incubated with 100 µL of the previously prepared PFC-NE and 100 µL phosphate buffer (see above), overnight at 4°C.

### Preparation of EP9-liposomes with luciferase-encoding mRNA (mRNA-LUC)

The ability to deliver potential therapeutic compounds to fibroblasts was evaluated by using modified messenger RNA (mRNA) encoding luciferase (mRNA-LUC) which was incorporated into EP9-decorated liposomes.

All oligos used in the following process to generate mRNA-LUC were synthesized by Integrated DNA Technologies (Coralville, IA, USA). The ORF/ CDS for *hRluc* (*Renilla reniformis*) was used and PCR reactions were performed with Phusion High-Fidelity DNA Polymerase with the following primers: hRluc fw: ATG GCT TCC AAG GTG TAC, hRluc rev: TCA AGC ATA GTC AGG TAC GTC ATA AGG GTA CTG CTC GTT CTT CAG CA. A phosphorylated forward primer was employed in the ORF PCRs to facilitate ligation to 5'UTR.

Splint ligations to add the 5'UTR and 3'UTR to the *hRLuc* CDS were performed with Ampligase Thermostable DNA Ligase (Epicenter Biotechnologies, Madison, WI, USA); 5'UTR plus HindIII: CTCACTAAGCTTAAATAAGAGAGAAAAGAAGAGTAAGAAGAAATATAAGAGCCACC GCT GCC TTC TGC GGG GCT TGC CTT CTG GCC ATG CCC TTC TTC TCT CCC TTG, 3'UTR plus ApaI: CAC CTG TAC CTC TTG GTC TTT GAA TAA AGC CTG AGT AGG AAG TGA GGG GGG CCC TCA CTC CGA.

UTR ligations were conducted in the presence of 200 nM UTR oligos and 100 nM splint oligos, with 5 cycles of the following annealing profile: 95°C for 10 s, 45°C for 1 min, 50°C for 1 min, 55°C for 1 min, 60°C for 1 min; hRluc-splint-5': CCT TGG AAG CCA TGG TGG CTC TTA TAT TTC TTC TT, hRluc-splint-3': CCC GCA GAA GGC AGC GTA CCT GAC TAT GCT TGA.

The fully ligated DNA construct was PCR-amplified before cloning into the vector (amplification fw: CTCACTAAGCTTAAATAAGAGAG, amplification rev: TCGGAGTGAGGGCC) and for cloning digested with HindIII and ApaI. The vector and inserts were ligated using a reaction mixture containing T4 DNA ligase and 10X T4 DNA ligase buffer. The ligation reaction was incubated for 16 h at 16°C. Ligation products were mixed with competent bacteria with a standard transformation protocol.

For the *in vitro* transcription, the construct was amplified from the vector by Phusion High-Fidelity DNA Polymerase (Thermo Fisher Scientific, Waltham, MA, USA) with a dT120 rev primer to achieve a 120 polyA tail (T7 fw: TAATACGACTCACTATAGGG, dtTail PCR rev: TTT TTT TTT TTT TTT TTT TTT TTT TTT TTT TTT TTT TTT TTT TTT TTT TTT TTT TTT TTT TTT TTT TTT TTT TTT TTT TTT TTT TTT TTT TTT TTT TTT TTT TTT TTT TTT TTT TTT TTT CCC TCA CTT CCT ACT CAG).

The IVT was performed using the mMESSAGE mMACHINE T7 ULTRA Transcription Kit (Thermo Fisher Scientific) resulting in the following transcript:

CTCACTAAGCTT AAATAAGAGAGAAAAGAAGAGTAAGAAGAAATATAAGAGCCACC

ATGGCTTCCAAGGTGTACGACCCCGAGCAACGCAAACGCATGATCACTGGGCCTCAGTGGTGGGCTCGCTGCAAGCAAATGAACGTGCTGGACTCCTTCATCAACTACTATGATTCCGAGAAGCACGCCGAGAACGCCGTGATTTTTCTGCATGGTAACGCTGCCTCCAGCTACCTGTGGAGGCACGTCGTGCCTCACATCGAGCCCGTGGCTAGATGCATCATCCCTGATCTGATCGGAATGGGTAAGTCCGGCAAGAGCGGGAATGGCTCATATCGCCTCCTGGATCACTACAAGTACCTCACCGCTTGGTTCGAGCTGCTGAACCTTCCAAAGAAAATCATCTTTGTGGGCCACGACTGGGGGGCTTGTCTGGCCTTTCACTACTCCTACGAGCACCAAGACAAGATCAAGGCCATCGTCCATGCTGAGAGTGTCGTGGACGTGATCGAGTCCTGGGACGAGTGGCCTGACATCGAGGAGGATATCGCCCTGATCAAGAGCGAAGAGGGCGAGAAAATGGTGCTTGAGAATAACTTCTTCGTCGAGACCATGCTCCCAAGCAAGATCATGCGGAAACTGGAGCCTGAGGAGTTCGCTGCCTACCTGGAGCCATTCAAGGAGAAGGGCGAGGTTAGACGGCCTACCCTCTCCTGGCCTCGCGAGATCCCTCTCGTTAAGGGAGGCAAGCCCGACGTCGTCCAGATTGTCCGCAACTACAACGCCTACCTTCGGGCCAGCGACGATCTGCCTAAGATGTTCATCGAGTCCGACCCTGGGTTCTTTTCCAACGCTATTGTCGAGGGAGCTAAGAAGTTCCCTAACACCGAGTTCGTGAAGGTGAAGGGCCTCCACTTCAGCCAGGAGGACGCTCCAGATGAAATGGGTAAGTACATCAAGAGCTTCGTGGAGCGCGTGCTGAAGAACGAGCAG

TAC CCT TAT GAC GTA CCT GAC TAT GCT TGA

GCT GCC TTC TGC GGG GCT TGC CTT CTG GCC ATG CCC TTC TTC TCT CCC TTG CAC CTG TAC CTC TTG GTC TTT GAA TAA AGC CTG AGT AGG AAG TGA GGGTTTTTTTTTTTTTTTTTTTTTTTTTTTTTT TTTTTTTTTTTTTTTTTTTTTTTTTTTTTT TTTTTTTTTTTTTTTTTTTTTTTTTTTTTT TTTTTTTTTTTTTTTTTTTTTTTTTTTTTT_._

The preparation of mRNA-LUC-loaded EP9-liposomes was performed by thin-film hydration technique as follows: L-α-phosphatidylcholine (soybean) (Avanti Polar Lipids, Alabaster, AL), 1,2‑dioleoyl-3-trimethylammonium-propane (DOTAP), cholesterol, DSPE-PEG_2000_, and DSPE‑PEG_2000_-maleimide were mixed in chloroform (at a ratio of 32.5, 32.5, 30.0, 4.75, 0.25% mol) until they were dissolved. Afterwards, the mixture was sonicated for 2 min and the chloroform was evaporated under a nitrogen stream. mRNA-LUC was dissolved in diethylpyrocarbonate (DEPC)-treated water at a concentration of 10 µg/mL. The thin film of lipids was hydrated with the mRNA-LUC solution and stirred using a magnetic stir bar for 1 h. Subsequently, the mixture was passed through a sterile filter (0.22 µm syringe filter).

For the targeting of the mRNA-LUC-loaded liposomes, they were incubated overnight at 4°C with synthesized peptides (EP9, MUT; see above) by mixing 100 µL of mRNA-LUC-loaded liposomes, 25 µg of peptide and 100 µL of DEPC-treated water. The targeted mRNA-LUC-loaded liposomes were stored at 4°C until further usage.

Human cardiac fibroblasts (HCF from PromoCell, Heidelberg, Germany) were seeded onto 24-well plates (1 x 10^5^ cells/well) and grown for 24 h. Thereafter, 100 µL of mRNA-LUC-loaded EP9-liposomes were added to each well and incubated at 37°C for 29 h. 5 min prior to analysis, 60 µM cell-permeable luciferase substrate ViviRen (Promega, Madison, WI, USA) was added. Luminescence was measured in an IVIS Lumina II *In Vivo* Imaging System (PerkinElmer, Waltham, MA, USA) for 3 min at different time points (3 h and 29 h) after addition of EP9-liposomes.

### Size distribution analysis of PFC-NE and liposomes

To determine the hydrodynamic diameter of PFC-NE and liposome preparations, 20 μL of the preparations were diluted in 980 μL of Milli-Q water and were analyzed by dynamic light scattering (DLS) (Nanotrac Wave II, Microtrac MRB, Haan, Germany). This technique also revealed the polydispersity index (PDI). The stability of PFC-NE was tested for up to two months at different pH values (pH: 4.5, 5.5, 6.5, and 7.4) by measuring particle size and PDI.

### *In vivo* imaging of infarcted hearts by MRI

To investigate whether cardiac stromal cells can be noninvasively visualized after MI using EP9-PFC-NE, we performed ^19^F-MRI as previously reported 20. For this purpose, 150 µL of EP9-PFC-NE were injected intravenously 5 days after MI (50 min ischemia/reperfusion). *In vivo* visualization by ^1^H/^19^F-MRI was carried out 24 h after EP9-PFC-NE injection on a Bruker 9.4 Tesla AVANCEIII wide bore NMR spectrometer (Bruker BioSpin, Ettlingen, Germany). ParaVision 5.1 was used to drive the instrument, which ran at frequencies of 400.21 MHz for ^1^H measurements and 376.54 MHz for ^19^F measurements. A 25 mm-quadrature ^19^F resonator with one channel adjustable to both ^1^H and ^19^F was used to collect the data. Mice were anesthetized with 1.5% (v/v) isoflurane and kept at 37°C. After morphological ^1^H pictures were acquired, the resonator was tuned to ^19^F, and anatomically corresponding ^19^F images were captured.

### Imaging of explanted hearts by MRI

For *ex vivo* ^1^H/^19^F-MRI allowing high-resolution imaging of post-MI hearts, we performed *in vivo* tissue fixation by cardiac perfusion with paraformaldehyde (4%). Mice were anesthetized with Thiopental. Thereafter, the thorax was opened to expose the heart, and the left ventricle was punctured with a needle connected to the perfusion solutions while a small incision was done in the right atrium for venous outflow. Hearts were first perfused with 10 mL of phosphate-buffered saline (PBS) with a speed of ~1 mL/10 s, followed by 3 mL of paraformaldehyde (4%). The explanted mouse hearts were kept in paraformaldehyde (4%) until further processing.

For ^1^H/^19^F-MRI, the fixed hearts were embedded in 1% agarose within a 2 mL vial, which was carefully fixed in the center of a 25 mm-birdcage resonator tunable to ^1^H or ^19^F. After acquisition of morphological ^1^H images, the resonator was tuned to ^19^F, and anatomically corresponding ^19^F images were recorded by using a RARE sequence (RARE factor 32, field of view: 2.56 cm^2^, matrix: 64 x 64, slices: 13, resulting in an in-plane pixel size of 0.4 x 0.4 mm^2^ after zero filling, slice thickness: 1 mm, repetition time, 7,500 ms, echo time: 6.98 ms, 2,500 averages, acquisition time: 10 h and 25 min).

### Transmission electron microscopy of cardiac tissue

To further explore the localization and cellular uptake of PFC-NE, we performed transmission electron microscopy (TEM). Sections of the explanted and paraformaldehyde-fixed hearts (see above) were cut into small pieces of approximately 1 mm^3^. Tissue samples were first incubated in 4% paraformaldehyde and 2% glutaraldehyde in 0.1 M cacodylate buffer, pH 7.4. at 4°C overnight, and then washed three times with 0.1 M cacodylate buffer. After that, the samples were treated with 1% osmium tetroxide for 60 min. The tissue blocks were dehydrated in a graded series of acetone, starting at 30% and increasing by 10% until reaching 100%. Samples were stained for 1 h in 1% uranyl acetate and 0.5% phosphotungstic acid in 70% acetone. The dehydration was completed and the samples were embedded in Spurr's medium for ultrathin sections (70 nm, Ultracut). After being stained with uranyl acetate and lead citrate, the slices were examined by electron microscopy (TEM Hitachi H7100, Tokyo, Japan) and digital images were acquired in high quality. From each of the samples multiple images (> 50 images) were taken and systematically analyzed for the occurrence of EP9-PFC-NE in phenotypically defined cell types.

### Isolation and cultivation of mouse cardiac fibroblasts and EpiSC

For cell isolation, hearts were harvested 5 days after MI (50 min ischemia/reperfusion). The isolation and cultivation of activated cardiac fibroblasts and EpiSC were performed by a Langendorff-based technique as previously described 4. In brief, hearts were rinsed with 3 mL (2 mL/min) of PBS via the coronary arteries, followed by digestion with collagenase type II (C2-BIOC, Merck, Darmstadt, Germany)-containing solution (1,200 U/mL) at 37 °C in ≤ 10 min. Digestion was performed by simultaneous perfusion with and immersion in collagenase solution under gentle rocking to obtain fibroblasts from the myocardium and EpiSC from the surface of the same heart.

The EpiSC-containing cell suspension collected from the heart surface was centrifuged at 55 × g for 2 min and the cell pellet containing cardiomyocytes was discarded. The supernatant was centrifuged at 300 x for 7 min and the cell pellet was resuspended in EpiSC culture medium (DMEM (high glucose; Sigma-Aldrich, Munich, Germany) with 30% fetal bovine serum (FBS; Biochrom, Berlin, Germany), 1% sodium pyruvate (100 mM; Invitrogen, Meerbusch, Germany), 1% penicillin-streptomycin (Biochrom)). EpiSC were plated in cell culture flasks (T75; Greiner Bio-One, Frickenhausen, Germany).

For the isolation of cardiac fibroblasts, collagenase-treated hearts (see above) were removed from the cannula and were further mechanically dissociated. The resulting cell suspension was collected, passed through a 100 μm cell strainer, and centrifuged at 55 × g for 1 min to remove cardiomyocytes. Afterwards, the supernatant was passed through a 40 μm cell strainer and centrifuged at 300 x g for 7 min to remove debris. The cell pellet was resuspended and magnetic bead depletion with Mojosort nanobeads (BioLegend, San Diego, CA, USA) was performed for CD31+ cells (endothelial cells) and CD45+ cells (immune cells). The remaining cells (fibroblasts) were resuspended in fibroblast culture medium [DMEM (high glucose) with 20% FBS, 1% penicillin-streptomycin, and plated on cell culture dishes. After 1 day of culture in an incubator at 37°C and 5% CO_2_ in a humidified atmosphere, cells were washed with PBS and fresh medium was added. For prolonged cultivation, the medium was replaced every 2 days, and cells were split at a confluence of about 80%.

### Analysis of peptide binding to different cell types by flow cytometry

To study the *in vitro* binding of 6-FAM-conjugated peptides (see above) we used the following cells: mouse EpiSC and cardiac fibroblasts prepared as described above, normal human dermal fibroblasts (NHDF) from juvenile foreskin, and human monocytes (THP-1). In addition, we used primary human cardiac fibroblasts which were prepared by outgrowth culture technique from a heart sample of a patient with terminal heart failure undergoing orthotopic heart transplant surgery, kindly provided by the Cardiac Surgery Department of the University Hospital Düsseldorf, Düsseldorf, Germany. A tissue sample weighing about ~5 g was removed from the failing heart's apex and placed in cooled BIOPS buffer to be divided into tiny pieces for subsequent culture to allow cell outgrowth in a 6‑well plate with DMEM (low glucose), 20% FBS, and 1% penicillin-streptomycin. The medium was replaced every 2 days and biopsy pieces were discarded after 5 days. After 14 days, cells reached a confluence of about 80% and were then passed to T75 cell culture flasks.

The binding assay was performed as follows: Cells were detached with PBS-EDTA (5 mM) for 10 min at 37°C and centrifuged at 500 × g for 5 min. Afterwards, 5 µg of synthesized peptide (see above) was added to 800 µL (0.5 x 10^6^ cells) of the cell suspension and incubated for 30 min at 37°C. To control the specific targeting of the EP9 peptide, scrambled and mutated peptides were used as negative controls. Subsequently, 200 µL of the cell suspension were washed in 2 mL MACS buffer (2 % FBS and 1 mM EDTA in PBS) and centrifuged at 500 × g for 5 min and the supernatant was discarded. One additional washing step was performed by resuspending the cell pellet in 200 µL of MACS buffer and centrifugation at 500 × g for 5 min. After discarding the supernatant, the cell pellet was resuspended in 200 µL of 4′6-diamidino-2-phenylindole dichlorhydrate (DAPI) (Merck, Darmstadt, Germany) (1 µg/mL) to allow discrimination of dead cells. Flow cytometric analysis was performed using a BD FACS Canto II flow cytometer (BD Biosciences, Franklin Lakes, NJ, USA).

### Ligand-receptor capture experiments using photoaffinity labeling and LC-MS/MS analysis

For the identification of potential binding partner proteins for EP9 on fibroblasts, we have synthesized a trifunctional molecule (diazirine photolinker), combining a maleimide moiety for ligand-coupling, a diazirine moiety for the receptor capture after the irradiation with UV light, and a biotin group for affinity purification using streptavidin (see Figure 5A). The biotin-labeled diazirine OSu ester was prepared according to a procedure by Müskens *et al.* 21 from 3-(4-(3-(trifluoromethyl)-3H-diazirin-3-yl)phenyl)propanoic acid 22 and 2-(2-(2-(2-azidoethoxy)ethoxy)ethoxy)ethan-1-amine 23. Before OSu ester formation, the free acid was purified by flash column chromatography on silica gel (dichloromethane/MeOH (15%)/acetic acid (2%)). From this, the OSu ester was obtained by HATU coupling followed by acidic workup as reported previously 21 and was used without further purification. The identity and purity of all compounds were controlled by spectroscopic analysis (NMR, ESI-MS).

The diazirine photolinker (1 mM) was coupled to EP9 (1 mM) by incubation for 1 h at room temperature to obtain the EP9 probe for the ligand-receptor capture (LRC) experiments. In parallel, the mutated peptide was used as a control ligand to control for unspecific binding (MUT probe). LRC experiments were performed according to the procedure by Müskens *et al*. 21 with modifications as follows: The experiment was made on NHDF cells that were grown in 150 mm-cell culture dishes with DMEM medium and 10% FBS to ≤ 80% confluence. Cells were washed three times with 5 mL PBS to remove the medium. The probe (EP9 probe or MUT probe) was diluted in Hank's balanced salt solution (HBSS) (Gibco, Thermo Fisher Scientific) to a final concentration of 1 µM and 6 mL of this dilution were added to the NHDF cell dishes. After incubation in the dark under constant agitation (Rocking Platform 444-0142; VWR, Radnor, PA, USA) for 30 min at 37°C, the cell dishes were placed on ice and exposed to 365 nm-100 W light (Everbeam, Surrey, CA, USA) for 30 min. For subsequent cell lysis, cells were washed with PBS and scraped with 1 mL/dish of lysis buffer (1X Protease inhibitor (Roche Diagnostics, Mannheim, Germany), 1% SDS in 50 mM Tris-HCl at pH 8). The lysates were then vortexed for 5 min and centrifuged at 16,000 × g for 5 min at 4°C. The supernatant was collected and frozen at -20°C until further use.

All following affinity purification steps were performed at 4°C and buffers were supplemented with cOmplete EDTA-free Protease Inhibitor Cocktail (Roche Diagnostics, Mannheim, Germany). The total volume of collected lysate from a dish was added to 250 µL of Dynabeads M‑280 Streptavidin (Invitrogen, Meerbusch, Germany), and the volume was filled up to 3 mL using PBS supplemented with 1% (v/v) NP40. The mixture of lysate and beads was incubated for 18 h in the dark at 4°C. Afterwards, the beads were washed four times with radioimmunoprecipitation assay (RIPA) buffer (Sigma-Aldrich), four times with PBS supplemented with 880 mM NaCl, and four times with PBS.

After pull down, the samples were washed sequentially with 1 mL PBS with 5% acetonitrile (ACN) and 1 mL HPLC-grade water, each for 5 min with end-over-end rotation at 4°C. The precipitated proteins were reduced, alkylated, and digested with 5 mM Tris(2-carboxyethyl) phosphine-hydrochloride (TCEP), 27.5 mM chloroacetamide (CAA), and 10 ng/µL Lys-C, respectively, in 8 M urea and 50 mM ammonium bicarbonate (ABC) for 3 h at room temperature. Afterwards, samples were diluted with ABC to a final urea concentration of 2 M and subjected to trypsin digestion overnight with a final concentration of 10 ng/µL in a thermo shaker (1,000 rpm at room temperature) to prevent settling of beads. Peptides were then desalted and stored on SDB-RPS (styrene-divinylbenzene resin) stage tips until LC-MS/MS analysis.

For LC-MS/MS analysis, peptides were eluted with 2% ammonium hydroxide and 80% ACN and dried in a vacuum concentrator. Dried peptides were resuspended in 2% ACN and 5% formic acid and analyzed on a Thermo QExactive PLUS instrument, coupled to a Thermo easy nLC 1000 instrument using a 60 min reverse phase elution gradient. Raw files were analyzed with MQ v2.0.1.0 using the Uniprot KB database including reviewed and unreviewed entries as well as protein isoforms. Further analysis was done in Perseus v1.6.14.0 and InstantClue v0.12.2.

### Modeling of CD63 complexes with peptides using ColabFold and AlphaFold 3

Models of human and mouse CD63 with EP9 were generated using a local installation of ColabFold 1.5.5 24 with AlphaFold Multimer 2.3 weights 25, and alignments generated by the MMSeqs2 26 API webserver. As suggested before for protein/peptide complexes 27, models were generated enabling the dropout layers and using 9 recycles, with default options otherwise. 200 random seeds were sampled, resulting in 1,000 models for each protein/peptide pair, using the weighted average of 0.8 ipTM + 0.2 pTM (interface predicted TM-score and predicted TM-score, respectively) as the confidence metric 25. Note that it has been recently shown that AlphaFold performs best for generating protein/peptide complexes compared to popular peptide docking software 27, and ipTM is the most suitable metric to discriminate peptide/receptor complexes among multiple state of the art measures 28. Additional models were generated using the EP9 sequence with the glycine spacer (GGGK(FAM)C) where the terminal cysteine was modified for a methionine residue to avoid spurious formation of disulfide bridges in the intracellular region (i.e., KLMLPRPGGGKM).

To generate potential alternative binding poses with AlphaFold 3 29, models were generated using the available webserver. Given the constraints imposed by the webserver, only two random seeds were tested for the human and mouse CD63‑EP9 complexes.

### CD63 expression analysis by flow cytometry

Cardiac cells were isolated by Langendorff-based digestion from hearts of infarcted and healthy mice as reported previously 4 and described above. The cell suspensions were centrifuged at 300 x g for 5 min and resuspended in MACS buffer with antibodies for PDGFR-α, CD31, CD45, and CD63 (Miltenyi Biotec, Bergisch Gladbach, Germany; diluted 1:100) and incubated for 20 min at 4°C. Samples were centrifuged at 300 x g for 5 min and the supernatant was discarded. To be able to discriminate dead cells, samples were stained with 1 μg/mL DAPI (Merck). Flow cytometric analysis was performed using a BD FACS Canto II flow cytometer.

### Single-cell RNA sequencing and CITE-seq data analysis

For comparison of gene expression among cardiac cell types after MI, a single-cell RNA sequencing (scRNAseq) data set of cardiac cells isolated from mouse hearts 5 days post-MI (50 min ischemia/reperfusion) previously published by us 30,31 was used. Heat maps were generated using Morpheus (https://software.broadinstitute.org/morpheus).

For analysis of CD63 and FAP protein expression in human hearts, a recently published CITE-seq (cellular indexing of transcriptomes and epitopes by sequencing) data set of healthy donor hearts, post-MI hearts, and hearts with cardiomyopathy 32 was used.

### Statistical information

Data are presented as means ± SD; n indicates the number of replicates (experiments, samples) as stated in the Figure Legends. Data were statistically analyzed using GraphPad Prism as stated in the Figure Legends. The threshold for statistical significance was set at *P* < 0.05.

## Results

### *In vivo* labeling of the left ventricle wall post-MI with EP9-PFC-NE

**Figure 2:**
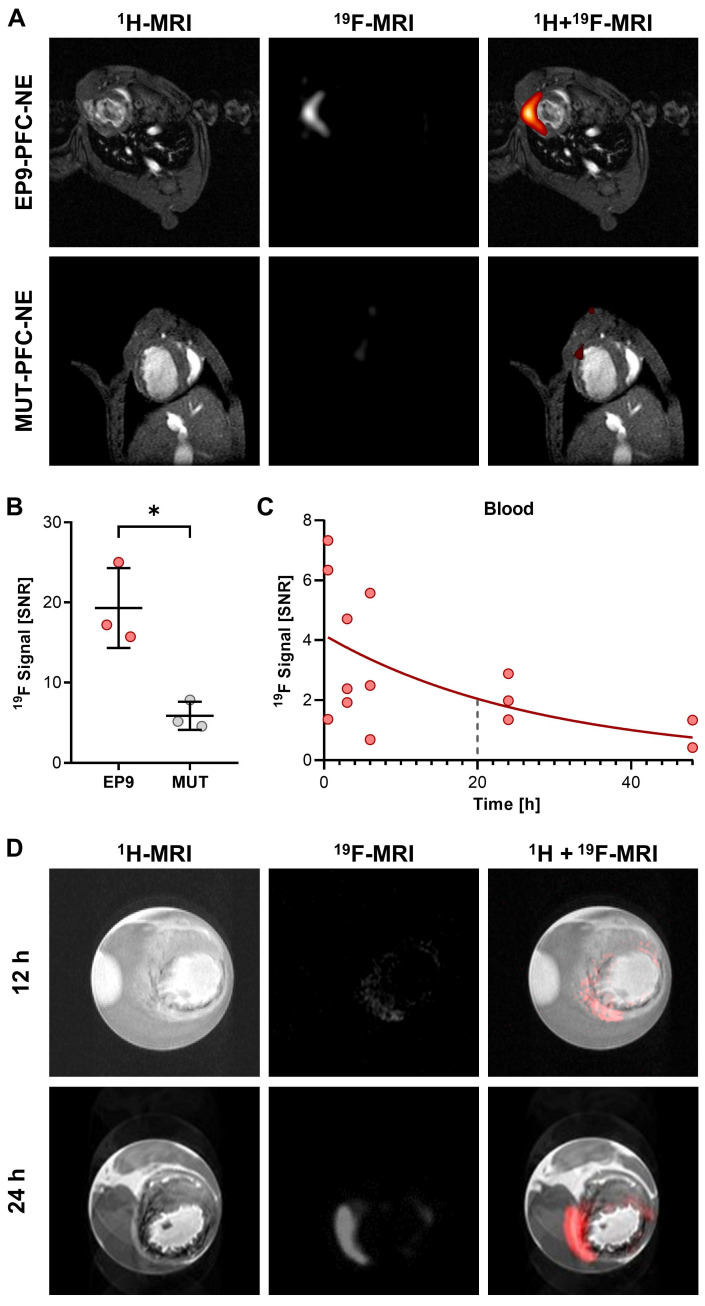
** Targeting of EP9-PFC-NE to the infarcted left ventricle wall visualized *in vivo* and *ex vivo* by ^1^H/^19^F MRI. EP9-PFC-NE (150 µL) was injected into the tail vein of mice 5 days after MI (50 min ischemia/ reperfusion).** For control, PFC-NE decorated with mutated peptide (MUT-PFC-NE, see Table S1) was used. **A:** Anatomically corresponding *in vivo* ^1^H and ^19^F MRI images from the mouse thorax recorded 24 h after PFC-NE injection. Representative images after EP9-PFC-NE injection (n = 3 mice) are shown in the upper row. Representative images after MUT-PFC-NE injection (n = 3 mice) are shown in the lower row. **B:** Quantification of ^19^F signal intensity in the left ventricle wall of the imaging data described in (A) (n = 3 each, unpaired t-test, *P < 0.05). **C:** Time course of the ^19^F signal intensity in circulating blood 0.5-48 h after intravenous injection of EP9-PFC-NE. The blue dashed line indicates the calculated half-life. SNR, signal-to-noise ratio. **D:** *Ex vivo* ^1^H and ^19^F MRI images of explanted and paraformaldehyde-fixed hearts harvested at 12 h and 24 h after EP9-PFC-NE injection.

In a previous study, we reported a high binding affinity of the peptide EP9 (Ac-KLMLPRPGGGKC-NH_2_) to rat epicardial stromal cells (EpiSC) formed after MI 17. In the present study, we first aimed to assess the potential of EP9 for *in vivo* imaging of epicardial and myocardial stromal cells (fibroblasts) in the post-MI mouse heart. To this end, we coupled EP9 to a ^19^F-containing PFC-NE (EP9-PFC-NE), designed for the *in vivo* labeling of cardiac cells (Figure 1). At 5 days post-MI (50 min ischemia/reperfusion), 150 µL of EP9-PFC-NE were injected into the tail vein of infarcted mice. PFC-NE coupled with a mutated version of the EP9 peptide (MUT-PFC-NE) served as a control. 24 h after EP9-PFC-NE or MUT-PFC-NE injection *in vivo* imaging by ^1^H/^19^F-MRI was performed. As shown in Figure 2A-B, a strong ^19^F signal was found to be closely associated with the infarcted area in the anterior wall left ventricle after EP9-PFC-NE injection. In contrast, the ^19^F signal in the MUT-PFC-NE control group was significantly smaller (Figure 2A-B) and was not selective for the left ventricle wall.

To investigate the temporal changes of the ^19^F signal following injection of EP9-PFC-NE, we measured the changes in the ^19^F signal in the blood after injection over time and also assessed the accumulation of ^19^F after explanting the hearts 12 h and 24 h after EP9-PFC-NE injection. As illustrated in Figure 2C, the ^19^F signal measured in blood after EP9-PFC-NE injection slowly declined with a calculated half-life of about 20 h. Representative ^1^H/^19^F images of explanted hearts, shown in Figure 2D, demonstrated that the ^19^F signal was transmurally distributed within the infarcted area of the left ventricle wall and increased over time (12 h and 24 h after EP9-PFC-NE injection). The extended availability of the EP9-PFC-NE in the bloodstream can explain the increase of the ^19^F signal in the infarcted heart over time (Figure 2D).

### Identification of cells within the infarcted left ventricle wall that preferentially take up EP9‑PFC‑NE

After having identified the infarcted left ventricle wall as a primary cardiac site of labeling, we next aimed to identify the cardiac cell type(s) that preferentially have taken up EP9-PFC-NE. To this end, transmission electron microscopy (TEM) was performed on the paraformaldehyde-fixed tissue samples to visualize by morphological criteria the location of the EP9-PFC-NE.

**Figure 3:**
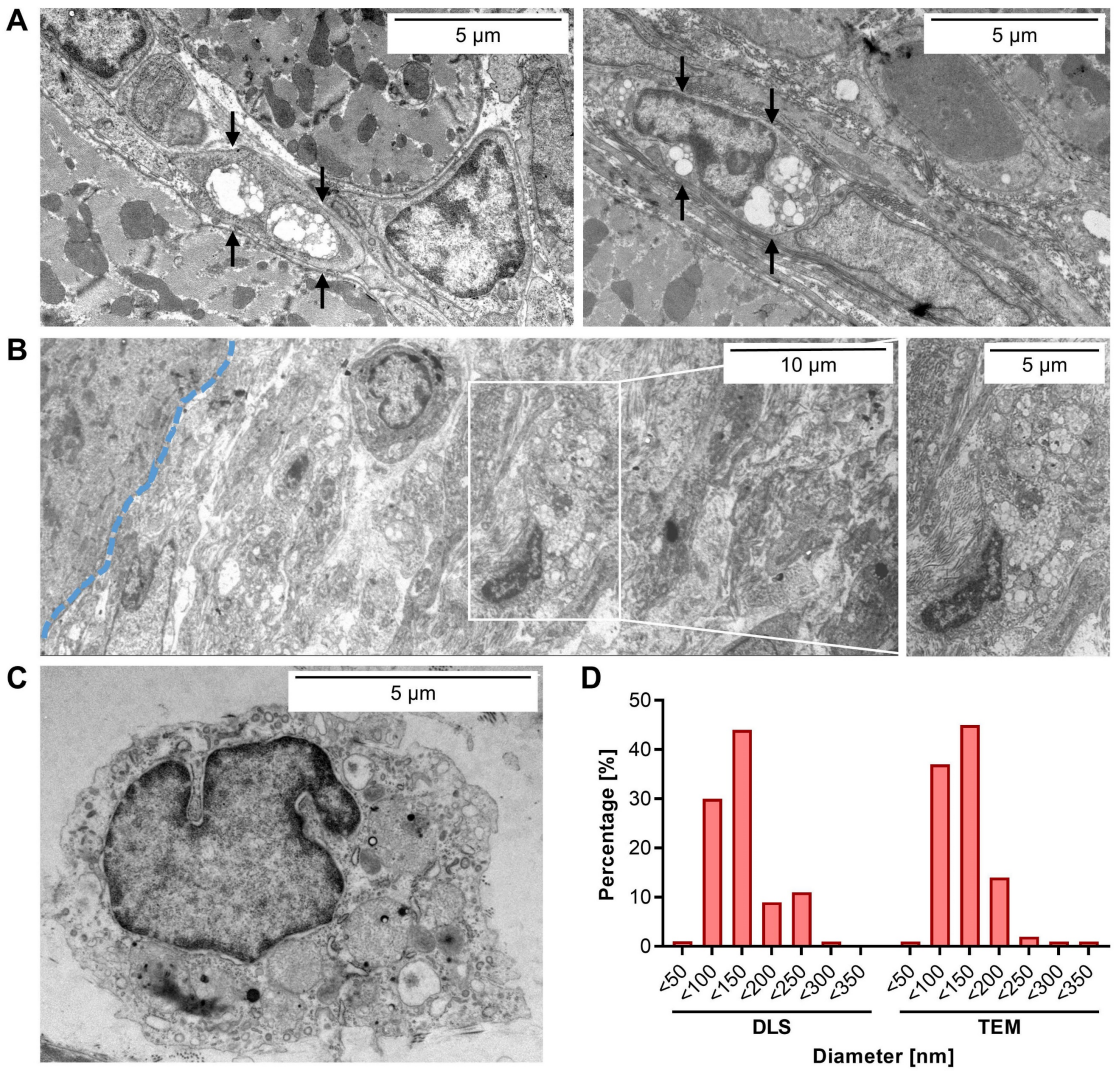
** EP9-PFC-NE uptake into cells of the infarcted left ventricle wall evaluated by transmission electron microscopy (TEM).** Tissue samples of the infarcted left ventricle wall harvested 6 days after MI were analyzed by TEM. EP9-PFC-NE was injected 1 day prior to sample harvest. Representative images (n = 3 hearts) are shown. **A:** Two images of fibroblasts containing vesicular structures (arrows) located in the interstitium between cardiomyocytes. For additional fibroblast images see Figure S3A. **B:** Image of EpiSC residing in the subepicardial layer with vesicular structures (blow-up). The blue dashed line indicates the border between cardiomyocytes (left) of the myocardium and the subepicardial EpiSC layer (right). **C:** Image of an immune cell. For additional images of immune cells see Figure S3B. **D:** Particle size distribution of EP9-PFC-NE prior injection as determined by dynamic light scattering (DLS) analysis (n = 3 measurements) and in the cardiac tissue after injection as quantified in the TEM images (n = 550 particles).

As shown in Figures 3A and S3, TEM images of the infarcted left ventricle wall revealed that in particular the elongated cells in the interstitial space between cardiomyocytes, phenotypically resembling cardiac fibroblasts, contained numerous intracellular electron-dense vesicular structures. The average size of these was measured to be 118 ± 47 nm (Figure 3D), which closely resembled the size distribution of EP9-PFC-NE determined by dynamic light scattering (DLS): 124 ± 49.2 nm (Figure 3D). We also found vesicles of similar size in the EpiSC constituting the subepicardial layer of the infarcted left ventricle wall (Figure 3B). In macrophages and cardiomyocytes of the left ventricle wall, we did not find vesicular structures (Figure 3A-C, Figure S3). We also found no evidence for the uptake of EP9-PFC-NE into endothelial cells of coronary vessels (Figure S4). Note, that PFC-NE particle integrity is maintained under acidic pH conditions resembling those in endosomes (~pH 6.0-6.5) or lysosomes (~pH 4.5-5.0) 33 for several days, as was assessed by DLS analysis (Figure S1). Together these observations indicate that EP9-PFC-NE were preferentially taken up by cardiac fibroblasts and EpiSC. Most likely these stromal cells are equipped with an EP9-binding receptor protein on their surface that triggers cellular uptake of EP9-PFC-NE.

### Binding of EP9 to different cell types

**Figure 4:**
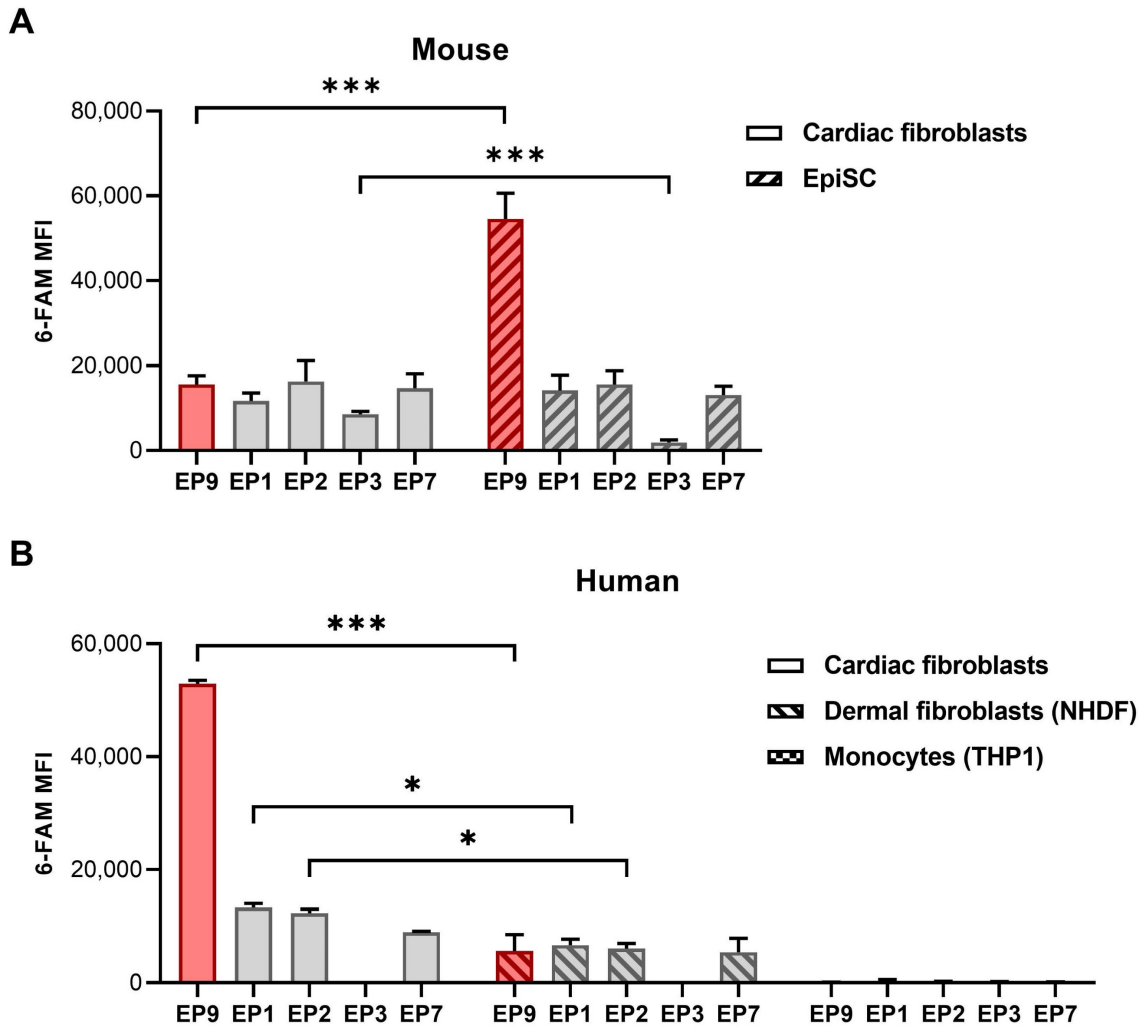
** Binding of peptide EP9 to different mouse and human cell types analyzed by flow cytometry.** The binding of EP9 in comparison to other stromal cell-targeting peptides (EP1, 2, 3, 7) recently identified 17 to different cell types was evaluated by flow cytometry after 30 min of incubation *in vitro*. **A:** 6-FAM fluorescence signal of the peptides at the surface of mouse cardiac fibroblasts isolated from healthy hearts and EpiSC isolated from hearts 5 days after MI (n = 3 hearts each). **B:** Human primary fibroblasts isolated from the explanted heart of a patient with terminal heart failure (n = 2 replicates), dermal fibroblasts (NHDF) (n = 3 replicates), and monocytes (THP1) (n = 3 replicates). Shown are means ±SD. Multiple t-tests with Holm-Sidak correction for multiple comparisons, ***P < 0.001, *P < 0.05. MFI, mean fluorescence intensity.

To further explore the binding of EP9 and other peptides previously identified to target rat cardiac stromal cells 17, we assessed the binding of these peptides to a broader spectrum of cell types derived from both mice and humans. As summarized in Figure 4A, all five small peptides bound to cultured cardiac fibroblasts and EpiSC from mice. Notably, EP9 showed superior binding to EpiSC compared to the other peptides, which is consistent with our previous findings in the rat heart 17. As illustrated in Figure 4B, EP9 displayed also high binding to primary human cardiac fibroblasts isolated (by outgrowth technique) from a heart sample of a patient with terminal heart failure undergoing heart transplant surgery.

Similarly, we found binding to human dermal fibroblasts (NHDF), albeit to a lesser extent, while no discernible binding was observed in human monocytes (THP1). The specificity of EP9 binding to mouse fibroblasts is supported by the strongly reduced binding of mutated and scrambled peptides (Figure S2); furthermore, only negligible binding to THP1 was observed (Figure S2).

### Identification of the cell surface receptor protein for EP9 by photoaffinity labeling

**Figure 5:**
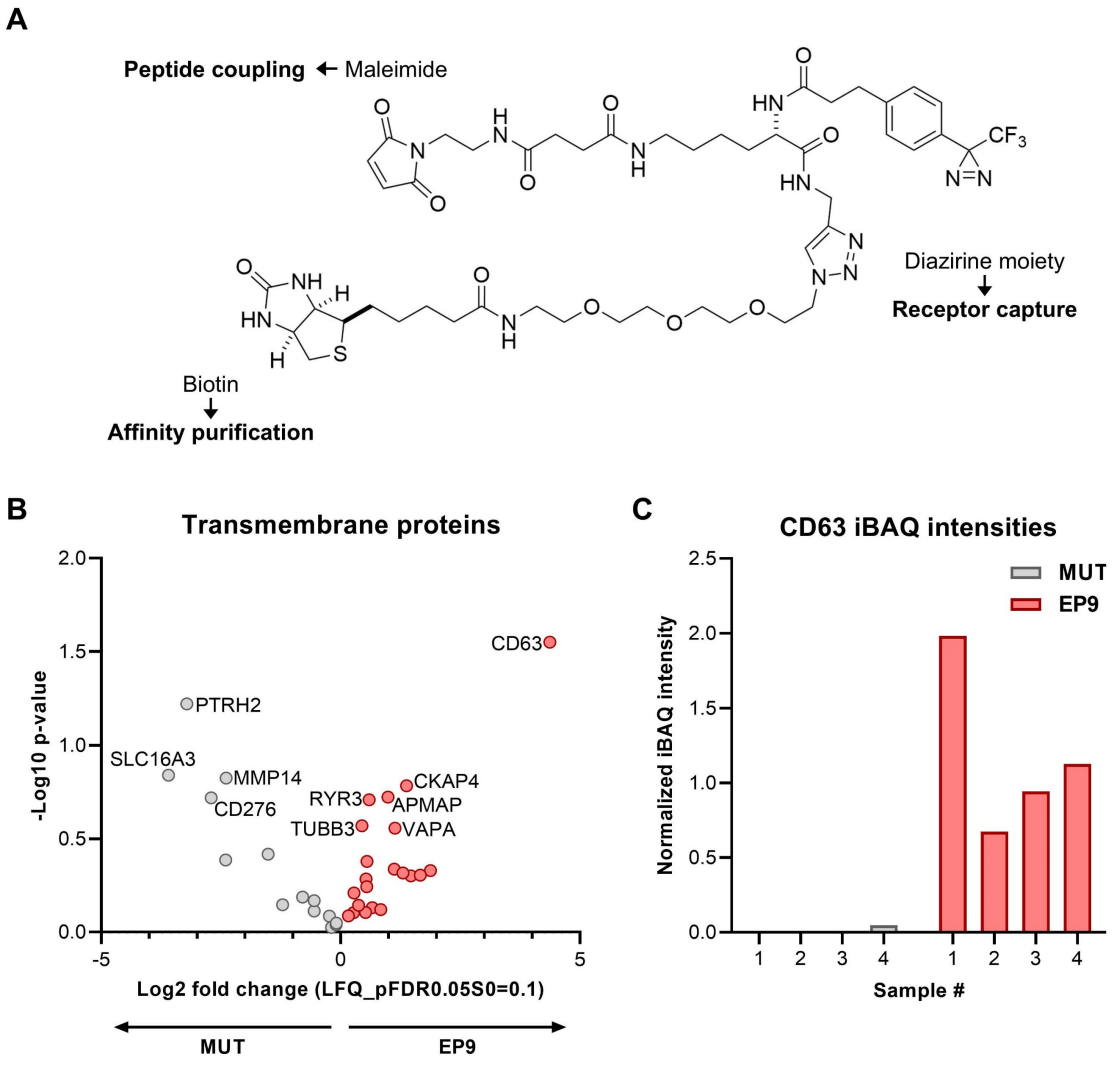
** Identification of the cell surface receptor protein for peptide EP9 in fibroblasts by photoaffinity labeling. A:** Chemical structure of the trifunctional probe used for the ligand-receptor-capture (LRC) experiments to identify the protein binding CD63. The probe contained a diazirine moiety for receptor capture upon UV light irradiation, maleimide for peptide coupling (see Figure 1B), and biotin for affinity purification by streptavidin-coated beads. **B:** The LRC experiment was performed by incubating human dermal fibroblasts (NHDF) with the trifunctional probe coupled to EP9 or the mutated peptide (MUT) for 30 min at 37°C (n = 4 samples each). After UV irradiation and protein affinity purification, captured proteins were identified by mass spectrometry (LC-MS/MS). The volcano plot shows identified transmembrane proteins (for the full protein list see Table S2) **C:** CD63 protein abundance as assessed by intensity-based absolute quantitation (iBAQ) in the LC-MS/MS samples from (B).

To identify the cellular receptor for EP9, most likely a cell surface protein, a ligand-receptor capture experiment (LRC) was performed using NHDF cells and a trifunctional probe, containing a diazirine photo linker, biotin for purification, and a maleimide reactive site for either coupling to EP9 or the mutated version of EP9 (MUT) as control (Figure 5A). By mass spectrometry, a total of 35 proteins were identified with the EP9 probe and/or the MUT probe. Comparison of the proteins captured by the EP9 probe and the MUT probe allowed the identification of proteins specifically bound by EP9. The volcano plot shown in Figure 5B lists the identified transmembrane proteins that were enriched in the EP9 or MUT probe samples. Notably, the tetraspanin CD63 emerged as the most significantly enriched protein in the EP9 probe samples (Figure 5B, C), while its signal was absent in the majority of MUT probe samples (Figure 5C). Other identified proteins include CKAP4 (cytoskeleton-associated protein 4) which was suggested to modulate fibroblast activation in the injured heart 34 and several less well-known proteins such as APMAP (Adipocyte Plasma Membrane-Associated Protein), TUBB3 (Class III beta-tubulin), VAPA (VAMP-associated protein A), and RYR3 (Ryanodine receptor 3). None of these proteins, however, displayed enrichment and significance values as high as those determined for CD63. Together our findings already suggest that CD63 is the primary candidate for the receptor of EP9.

**Figure 6:**
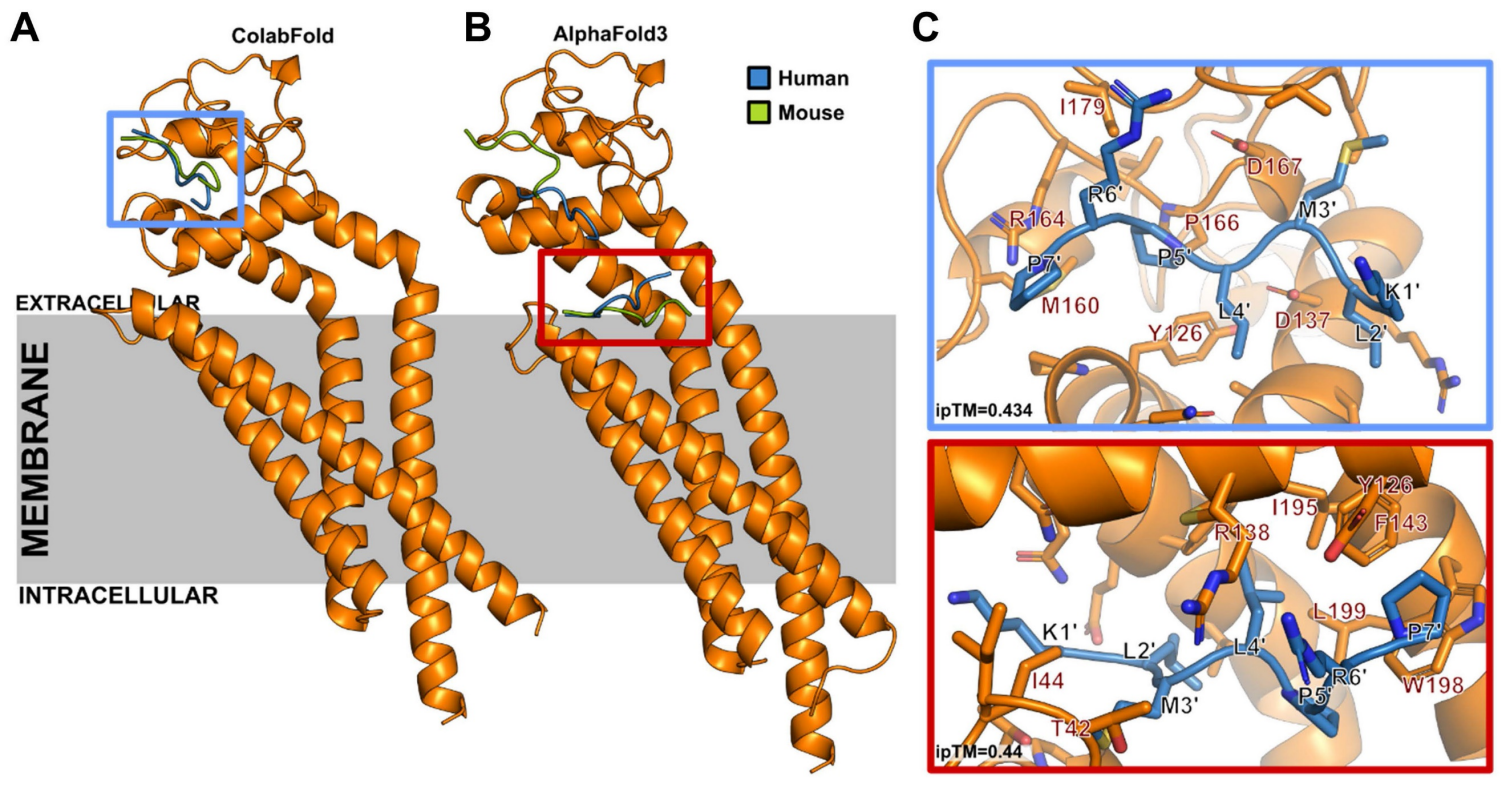
** Human and mouse CD63-EP9 complexes as modeled with ColabFold and AlphaFold3.** Models of the complex formed by EP9 and human or mouse CD63 were generated with a local ColabFold installation and the AlphaFold 3 webserver. **A:** From 1,000 models generated with ColabFold for human and mouse CD63 with EP9, all models located EP9 in the extracellular region in a groove of the large extracellular loop (EC2). Only the top-ranked poses are shown for both cases (blue, human CD63; green, mouse CD63). **B**: The pose top-ranked by AlphaFold 3 is located at the extracellular end of the central cavity, common to tetraspanins. Lower-ranked models show interactions with the same groove as that shown in ColabFold models. In both (A) and (B) only the human CD63 structure is shown for simplicity. **C:** Zoom into the binding site of ColabFold (top) and AlphaFold 3 (bottom) predicted top-ranked poses. ipTM, interface predicted TM-score.

In order to characterize the binding site of EP9 to CD63 we carried out molecular modeling studies of human and mouse EP9-CD63 complexes using ColabFold 24 and AlphaFold 3 29. Both methods yield ipTM scores of 0.434 and 0.44, respectively. Scores in this range have been found for known receptor-peptide complexes before 28. As summarized in Figure 6 and Video S1, all models located binding of EP9 to the CD63 extracellular region within a groove of the large extracellular loop (EC2). Similar results were obtained when CD63-EP9-glycine spacer complexes were modeled (Figure S5). The pose top-ranked by AlphaFold 3 is located at the extracellular end of the CD63 central cavity. Together these findings indicate that EP9 specifically binds to the extracellular loop of CD63 in both mice and humans.

**Figure 7:**
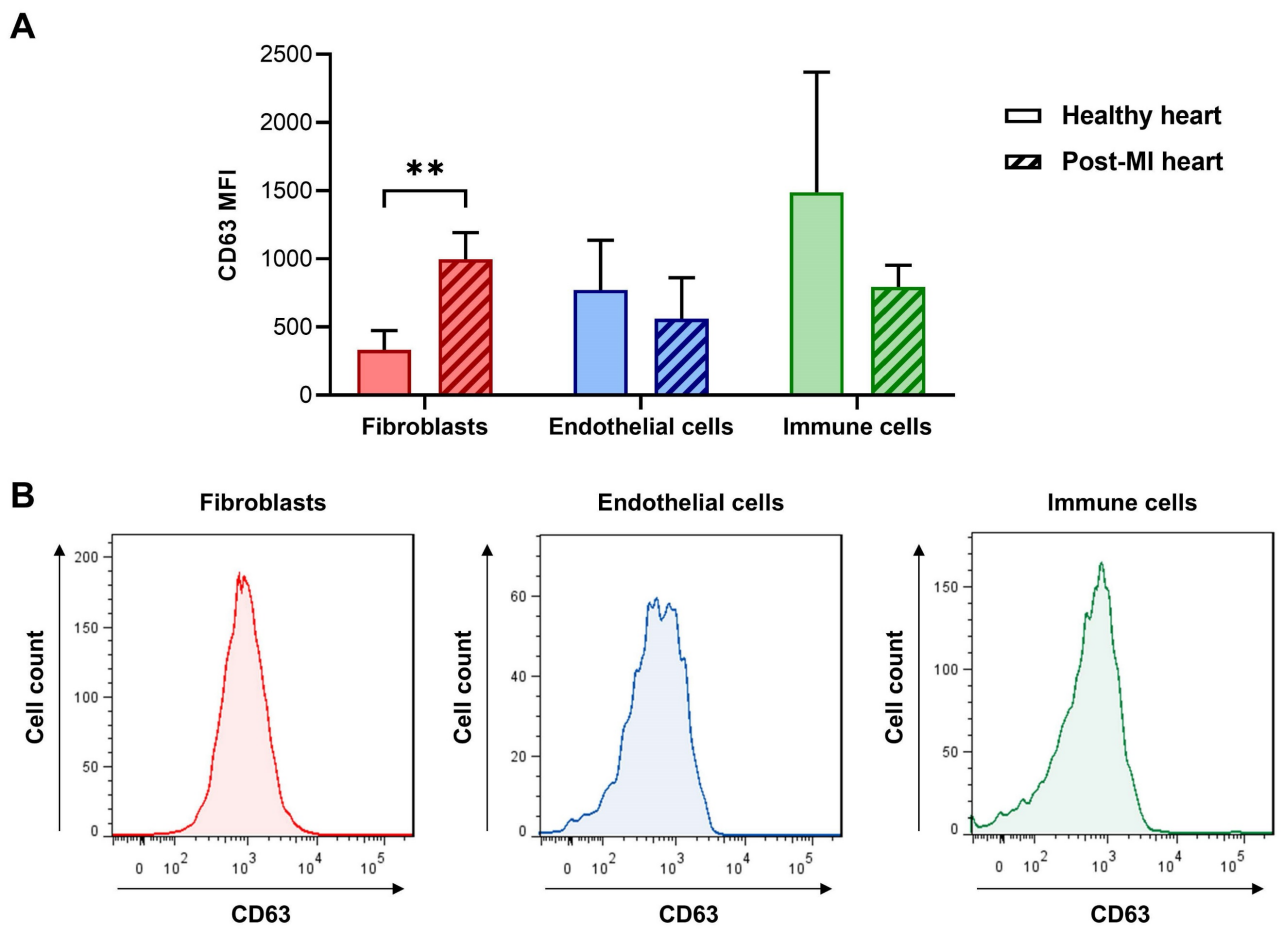
** CD63 expression at the surface of freshly isolated cardiac cells evaluated by flow cytometry. A:** Cardiac fibroblasts, immune cells, and endothelial cells were isolated from non-infarcted (healthy) and infarcted hearts (50 min ischemia/reperfusion) 5 days after MI and analyzed by flow cytometry (n = 6 cell preparations from infarcted hearts and n = 4 cell preparations from healthy hearts, multiple t-tests with Holm-Sidak correction for multiple comparisons, **P < 0.01). For the gating strategy of cardiac fibroblasts (PDGFR-α+/ CD31-/CD45-), immune cells (CD45+), and endothelial cells (CD31+) see Figure S6. **B:** Representative histograms of the post-MI data shown in (A).

To study the expression of CD63 in cardiac fibroblasts, endothelial cells, and immune cells we used flow cytometry on the various cell types isolated from infarcted and healthy (control) mouse hearts. As shown in Figure 7A, CD63 was significantly increased in cardiac fibroblasts after myocardial infarction, while there was no difference between MI and control samples of endothelial cells and cardiac immune cells. It should be noted, that in collagenase-perfused hearts it is technically not possible to selectively isolate infarct-activated cardiac fibroblasts from the cardiac fibroblasts of the non-infarcted tissue 4, resulting in a mixture of activated and non-activated cardiac fibroblasts. Therefore, the true differences between control fibroblasts and MI-activated fibroblasts (Figure 7A) are likely to be larger.

### EP9-decorated liposomes as a drug delivery system

**Figure 8:**
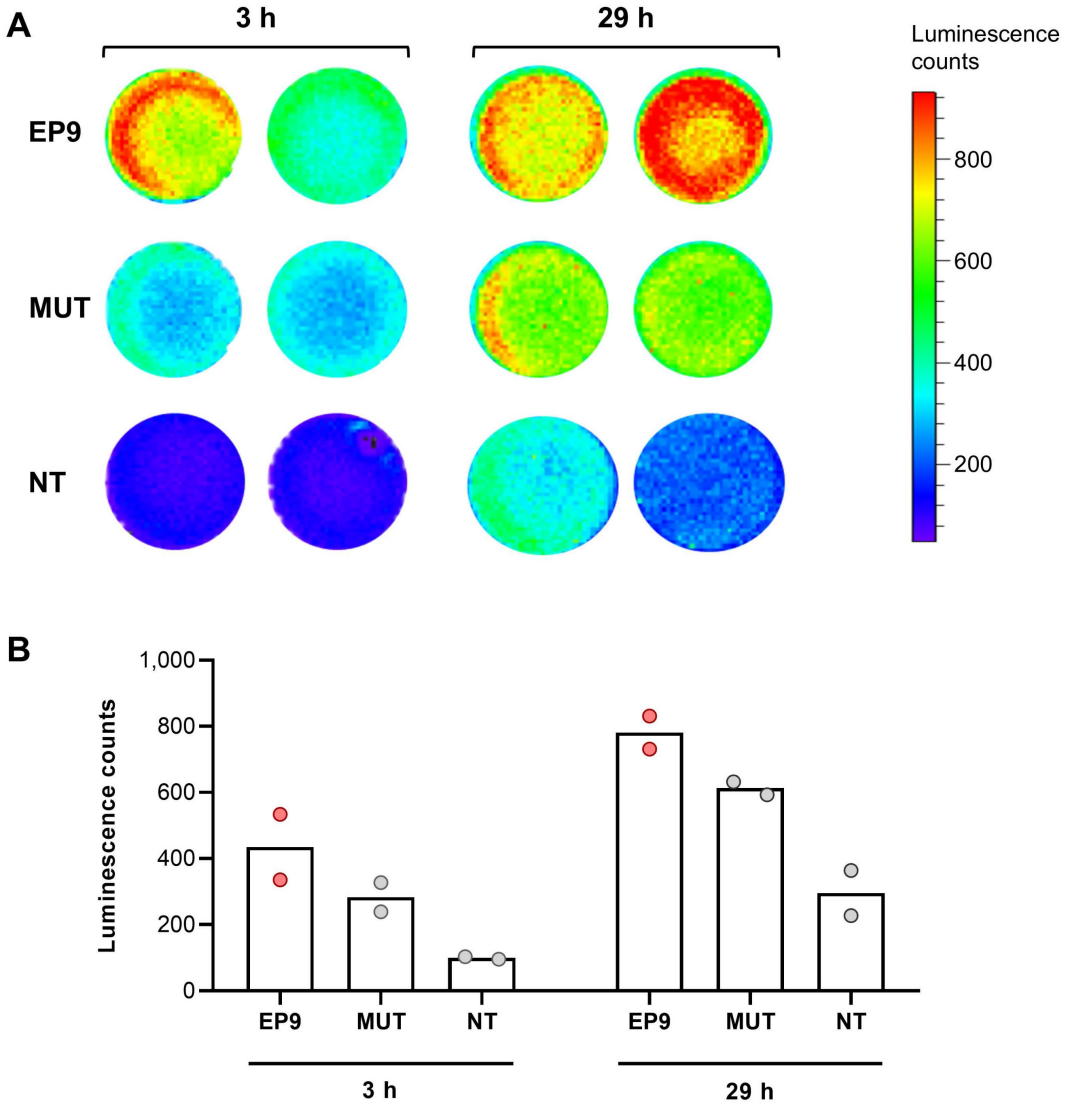
** Uptake and expression of luciferase-encoding mRNA (mRNA-LUC) incorporated in EP9-decorated liposomes into human cardiac fibroblasts analyzed by luminescence imaging.** EP9-liposomes were loaded with luciferase-encoding mRNA (mRNA-LUC). For control, mRNA-LUC was loaded into liposomes decorated with the mutated peptide (MUT) or non-targeted liposomes without peptide (NT). Human cardiac fibroblasts (PromoCell HCF) were seeded into a 24-well plate and confluency was controlled by light microscopy. After 3 h and 29 h incubation with the different liposome preparations, cell-permeable luciferase substrate was added and emitted luminescence was measured using an IVIS imaging system. **A:** Images of luminescence signals (n = 2 technical replicates). **B:** Quantification of luminescence signal counts of samples shown in (A).

To explore the utility of EP9-decorated liposomes for the delivery of therapeutic compounds to cardiac fibroblasts, we loaded EP9-liposomes with modified mRNA coding for luciferase (mRNA-LUC). Luciferase was chosen because only when mRNA is taken up by the cells and is intracellularly transcribed into the protein, light is emitted that can be measured in real-time by sensitive luminometry. As shown in Figure 8, incubation of human cardiac fibroblasts with EP9-liposomes containing mRNA-LUC resulted in a clear luminescence signal which increased over time. The light signal was weaker when using mutated peptide (MUT)-decorated or non-targeted (NT) liposomes. These finding clearly demonstrates that binding of EP9 to its receptor, most likely CD63, is a prerequisite for targeting the nanoemulsions or liposomes to fibroblasts and the subsequent cellular uptake.

## Discussion

This study reports the visualization of infarct-activated stromal cells (cardiac fibroblasts and epicardial cells) with ^19^F-MRI using the peptide-decorated nanoemulsion EP9-PFC-NE. In addition, we have identified CD63 as a novel target on activated fibroblasts to which our previously published targeting peptide EP9 17 binds. Given the molecular features of fibroblast CD63, this might open new possibilities for *in vivo* imaging as well as targeted delivery of therapeutic compounds.

CD63 belongs to the superfamily of tetraspanins which are cell surface-associated membrane proteins characterized by four membrane domains 35 and a large extracellular loop (EC2) that caps the central transmembrane cavity formed by the transmembrane helices 36. Models generated with ColabFold 24 and AlphaFold 3 29 suggest that EP9, with or without the linker, can bind to human or mouse CD63 in a groove located in EC2, or in the extracellular end of the central cavity (Figures 6 and S5). The extracellular side is accessible for the peptide at the cellular surface and shows the highest level of sequence variability among tetraspanins 37, which might explain the high specificity of EP9 for CD63.

A main function of CD63 is believed to enable the internalization of partner proteins 38. Mechanistically, the C-terminus of CD63 contains a tyrosine-based internalization motif that confers a fast rate of endocytosis as well as a prominent localization in late endosomes 38. In line with this known function, we observed a massive accumulation of vesicular structures resembling EP9-PFC-NE only within cardiac fibroblasts and epicardial cells (EpiSC) after injection (Figures 3A, S2A).

Most likely the cellular uptake is a two-step process: After binding of EP9-PFC-NE to CD63, the peptide and its associated cargo is internalized to accumulate intracellularly. Consistent with this interpretation is the observation that the *in vivo* plasma half-life of EP9-PFC-NE is as long as 20 h after intravenous injection so that sufficient time is available for continuous cellular binding and uptake. This feature can explain our finding that the ^19^F signal within the infarcted heart increased over time (Figure 2D) when the nanoemulsion was systemically applied.

Using a yeast two-hybrid approach, the main endogenous binding partner of CD63 - aside from the β1 integrin subunit - has been identified as TIMP-1 (TIMP metallopeptidase inhibitor 1), and TIMP-1 binding to CD63 was reported to inhibit cell growth and apoptosis 39. TIMPs are well known to maintain the homeostatic balance of the extracellular matrix by inhibiting activated matrix 39. Interestingly, enhanced expression of CD63 was recently reported in cardiac fibroblasts after TAC 40, which is similar to our findings after MI (Figure 7A). In addition, this study reported the promotion of myocardial fibrosis through an interaction between CD63 and integrin β1 on cardiac fibroblasts 40. Therefore, CD63 could also be a potential target in treating fibrosis. Interestingly, iron can induce CD63 expression 41, and treatment with intravenous iron in a clinical setting is known to reduce both cardiovascular death and heart failure-related events in a broad population 42. Whether CD63 is causally involved in iron-mediated cardiac pathologies remains to be elucidated.

Electron microscopy clearly identified cardiac fibroblasts and EpiSC as the major cell types incorporating EP9-PFC-NE under *in vivo* conditions (Figure 3A, B). This finding contrasts with flow cytometry data showing expression of CD63 also on endothelial cells and cardiac immune cells (Figure 7A). Obviously, the mere presence of CD63 is not necessarily associated with cellular uptake. Interestingly, CD63 has been reported in fibroblasts to be associated with clathrin-coated pits and caveolae, the latter two known to mediate endocytosis 35. Furthermore, treatment of fibroblast with CD63 antibodies found the antibodies internalized in caveolae-like structures 35. We therefore hypothesize that fibroblast uptake of EP9-PFC-NE involves aside binding of EP9 to CD63 the cellular uptake via caveolae and/or clathrin-coated pits. In support of a two-step process is our observation that the expression of mRNA-LUC was attenuated when a mutated peptide was used (Figure 8). In addition, a reanalysis of recently published scRNAseq data of the infarcted heart 30 revealed that genes coding for caveolae proteins are predominantly expressed in cardiac fibroblasts and epicardial cells/EpiSC (Figure S7). On the other hand, genes coding for clathrin-coated pits show a broader expression profile with a certain preference for immune cells and endothelial cells (Figure S7). It also should be noted that posttranslational modification CD63 by N-glycosylation 43 may constitute an additional regulatory mechanism.

Cardiac fibrosis is a common feature of various cardiomyopathies including MI which unfavorably alters cardiac structure and function and can lead to heart failure 44. Specific cardiac antifibrotic therapies are lacking, making cardiac fibrosis an urgent unmet medical need 45. A fibroblast-targeted nanoemulsion system may fill this gap. The present study used mRNA-LUC to show the general feasibility of this approach for the future development of RNA-based therapeutics to treat cardiac fibrosis. Alternatively, liposomes are well suited to deliver low molecular weight substances leading to sustained exposure of the targeted region. This approach has been actively pursued in the tumor field and several clinical trials are under way 46. In the cardiovascular system, targeting individual cell types such as macrophages, cells of the coronary vasculature, and cardiomyocytes with nanoemulsions so far did not result in major clinical studies. In summary, our study opens the intriguing possibility of developing novel antifibrotic therapies by loading EP9-targeted liposomes with low molecular weight drugs, peptides, proteins, and/or nucleic acids.

FAP is expressed by cancer-associated fibroblasts but also by activated cardiac fibroblasts early after acute MI in patients 47. Several radiolabeled FAP inhibitors (FAPI) have been developed for molecular imaging by PET and potential theranostic applications 48. Both FAP and CD63 appear to have a similar cellular distribution, but this does not imply that their response to pathophysiological stimuli is similar. When comparing gene expression of the two membrane proteins using scRNAseq data sets recently published by us on cardiac cells 30,31, CD63 is mainly expressed in the various fibroblast subpopulations and epicardial cells/EpiSC (Figure S8) but is also found on immune cells and endothelial cells consistent with our expression analysis by flow cytometry (Figure 7). Note that the highest expression was in myofibroblasts (CF_2). In comparison, FAP as expected showed strong expression in all cardiac fibroblast subpopulations, with little expression in epicardial cells/EpiSC. In general, the expression of CD63 was about 50-fold higher as compared to FAP (Figure S8). A similar picture can be obtained by reanalysis of recently published data on the protein expression of CD63 and FAP in the human heart 32. As shown in Figure S9, CD63 again is highly expressed in the cardiac fibroblast fraction and exceeds the protein expression of FAP. Remarkably, CD63 in cardiac fibroblasts was significantly increased in the acutely infarcted heart and other cardiac pathologies, while changes in FAP were generally less pronounced (Figure S9B). Together these data suggest, that CD63-mediated nanoparticle uptake in activated cardiac fibroblasts may have theranostic use in humans.

In summary, this study has identified CD63 on infarct-activated fibroblasts and EpiSC as the main *in vivo* binding partner for EP9-PFC-NE, which is followed by cellular uptake. Thus, EP9-decorated compounds may offer new diagnostic and therapeutic potential in the evaluation and treatment of cardiac fibrosis.

## Supplementary Material

Supplementary figures and tables.

Video S1.

## Abbreviations

MI: myocardial infarction
EpiSC: epicardium-derived stromal cells
PFC-NE: perfluorocarbon-containing nanoemulsions
EMT: epithelial-to-mesenchymal transition
DLS: dynamic light scattering
TEM: transmission electron microscopy
MRI: magnetic resonance imaging
PET: positron emission computed tomography
LAD: left anterior descending coronary artery
PDI: polydispersity index
NHDF: Normal Human Dermal Fibroblasts
FAP: fibroblast activation protein
6-FAM: carboxyfluorescein
DSPE-PEG_2000_-maleimide: 1,2-distearoyl-sn-glycero-3-phosphoethanolamine-N-[maleimide(polyethyleneglycol)-2000]
PBS: phosphate-buffered saline
DOTAP: 1,2-dioleoyl-3-trimethylammonium-propane
DEPC: diethylpyrocarbonate
LRC: ligand receptor capture
FBS: fetal bovine serum
DAPI: 4′6-diamidino-2-phenylindole dichlorhydrate
HBSS: Hank's balanced salt solution
TCEP: Tris(2-carboxyethyl) phosphine-hydrochloride
CAA: chloroacetamide
ABC: ammonium bicarbonate
scRNAseq: single-cell RNA sequencing
CITE-seq: cellular indexing of transcriptomes and epitopes by sequencing
ipTM: interface predicted TM-score
TIMP-1: TIMP metallopeptidase inhibitor 1
TAC: transverse aortic constriction
FAPI: fibroblast activation protein inhibitor
iBAQ: intensity-based absolute quantitation
