# Supplementary material for: CD63 as novel target for nanoemulsion-based 19F MRI imaging and drug delivery to activated cardiac fibroblasts
Source: Theranostics. 2025 Jan 1;15(1):1–18. doi: 10.7150/thno.96990 (PMC11667234; doi:10.7150/thno.96990)
Supplement: Supplementary file 1 — Supplementary figures and tables. [file thnov15p0001s1.pdf]

Supplementary items

Figures S1-S9

Tables S1-S2

Video S1

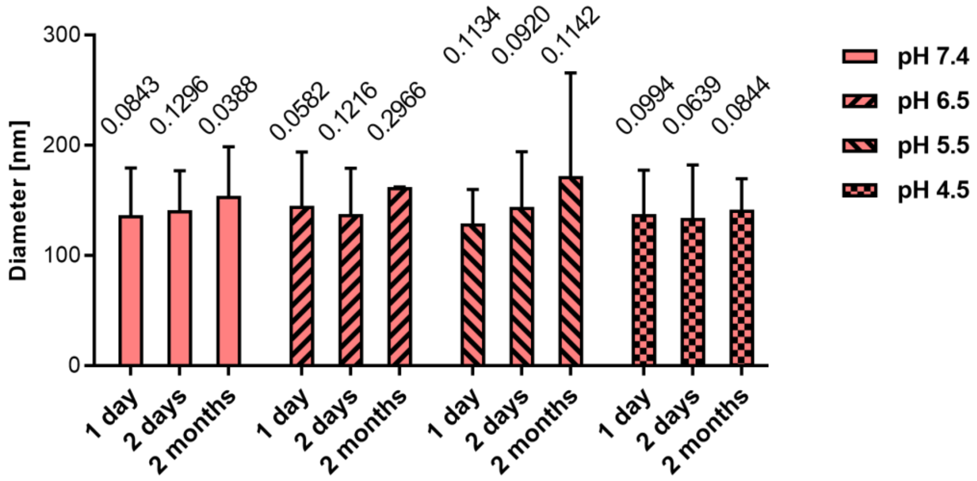

Figure S1. Stability of PFC-NE at acidic pH.

Average size (diameter) of PFC-NE after incubation at acidic pH conditions for 1 day, 2 days, and 2 months at 4°C as determined by dynamic light scattering (DLS) analysis (n = 3 measurements). The values above each bar report the polydispersity index (PDI), a measure of particle homogeneity.

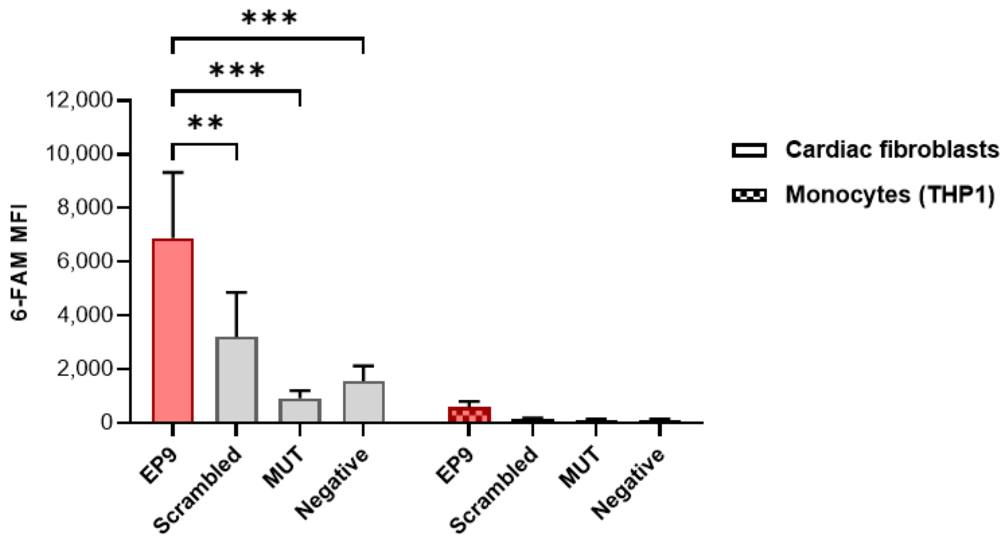

Figure S2. Binding of peptide EP9 in comparison to control peptides to mouse cardiac fibroblasts and human monocytes (THP1).

The binding of peptide EP9 in comparison to EP9-derived scrambled and mutated (MUT) peptides (see Table S1), as well as a negative control peptide (Negative) with low affinity to cardiac stromal cells (as previously assessed [17]: peptide EP50), was evaluated by flow cytometry after 30 min of incubation in vitro. 6-FAM fluorescence signal of the peptides at the surface of mouse cardiac fibroblasts isolated from hearts 5 days after MI (n = 3 hearts) and human monocytes (THP1) (n = 3 replicates). Two-way ANOVA with Sidak's multiple comparisons test, ***P* < 0.01, ****P* < 0.001.

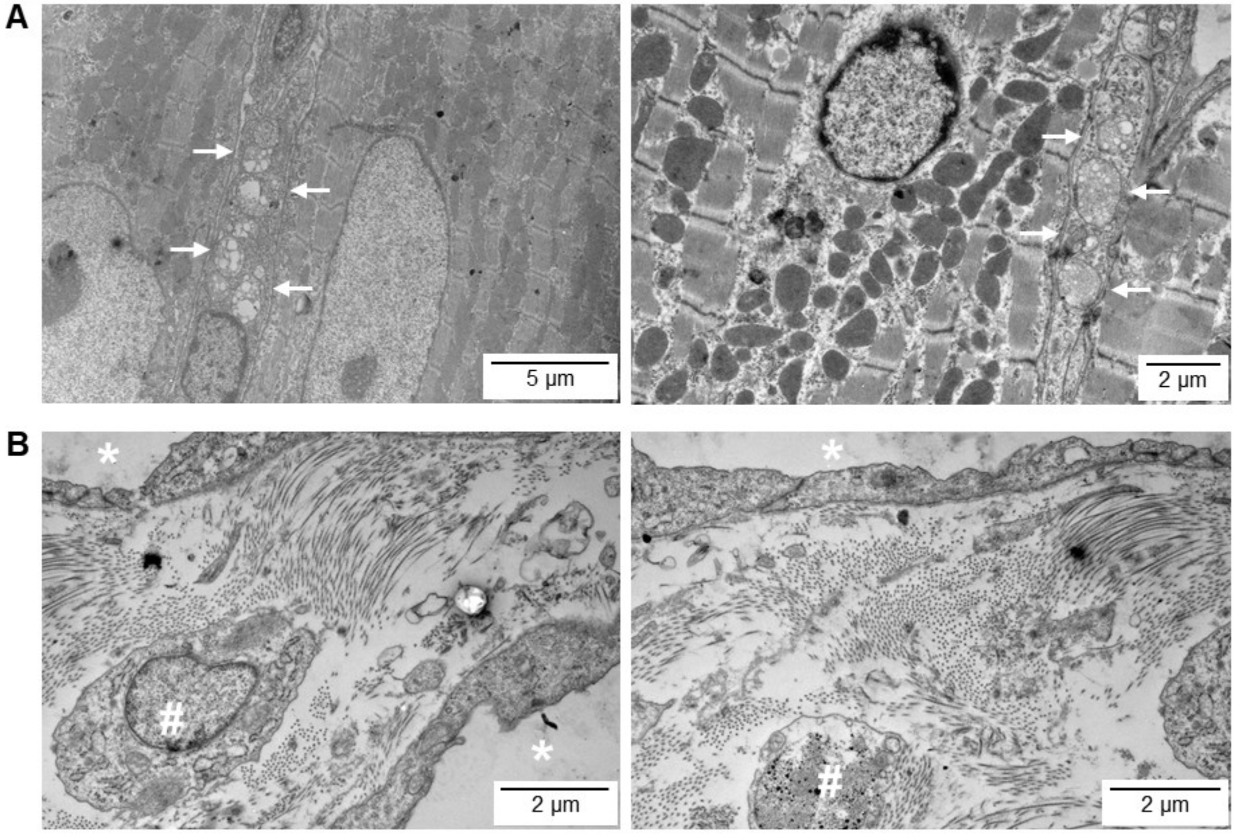

Figure S3. Additional images of EP9-PFC-NE uptake into cells of the infarcted left ventricle wall evaluated by transmission electron microscopy (TEM).

Additional images of the samples described in Figure 3. **A:** Images of fibroblasts containing vesicular structures (arrows) located in the interstitium between cardiomyocytes. **B:** Images of immune cells (#). Asterisks label capillaries/vessels.

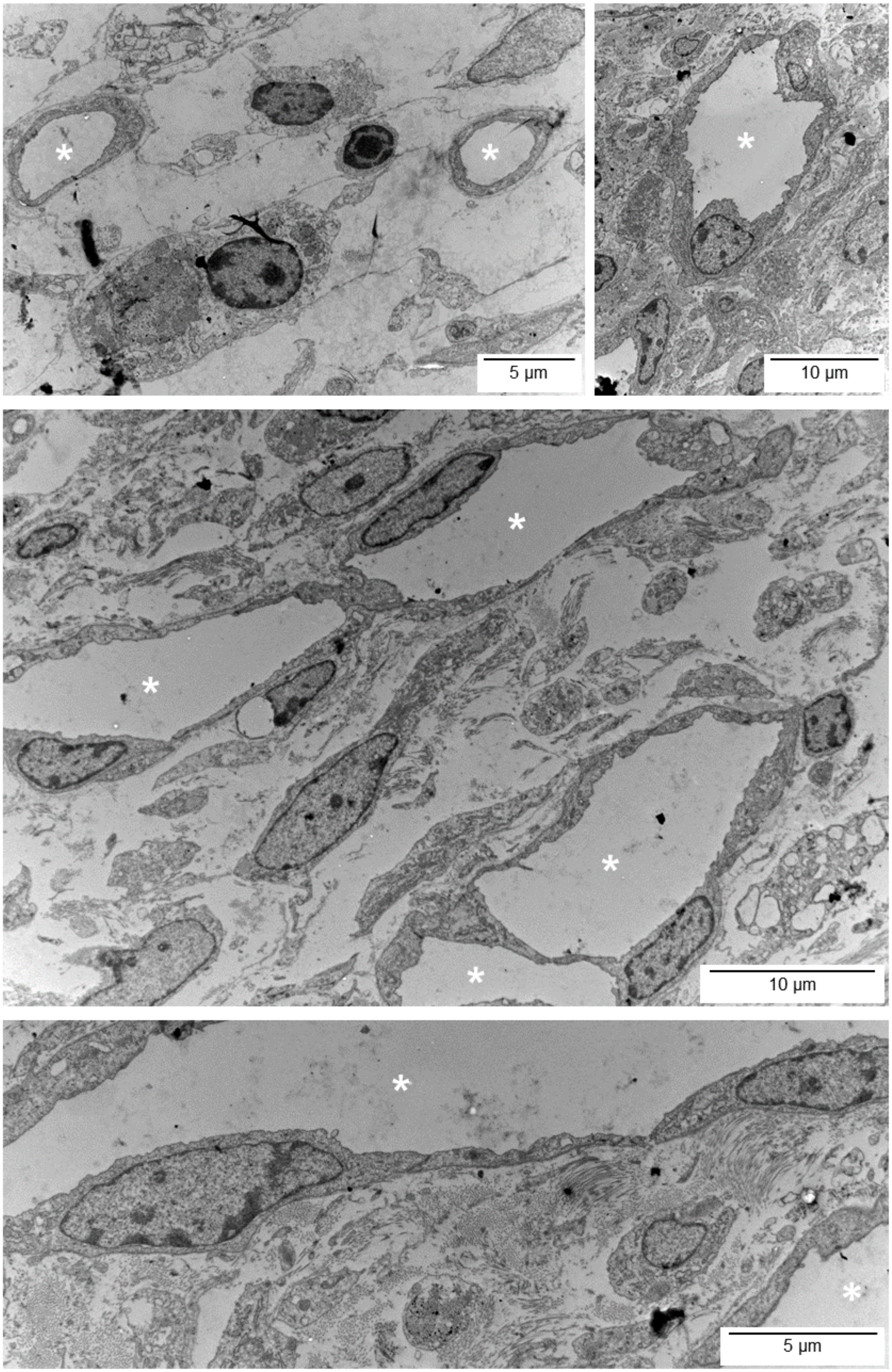
5 µm
10 µm
10 µm
5 µm

Figure S4. Images of cardiac endothelial cells of the infarcted left ventricle wall after **EP9-PFC-NE injection evaluated by transmission electron microscopy (TEM)**.

Images of the samples described in Figure 3 showing endothelial cells of capillaries and larger vessels (asterisks).

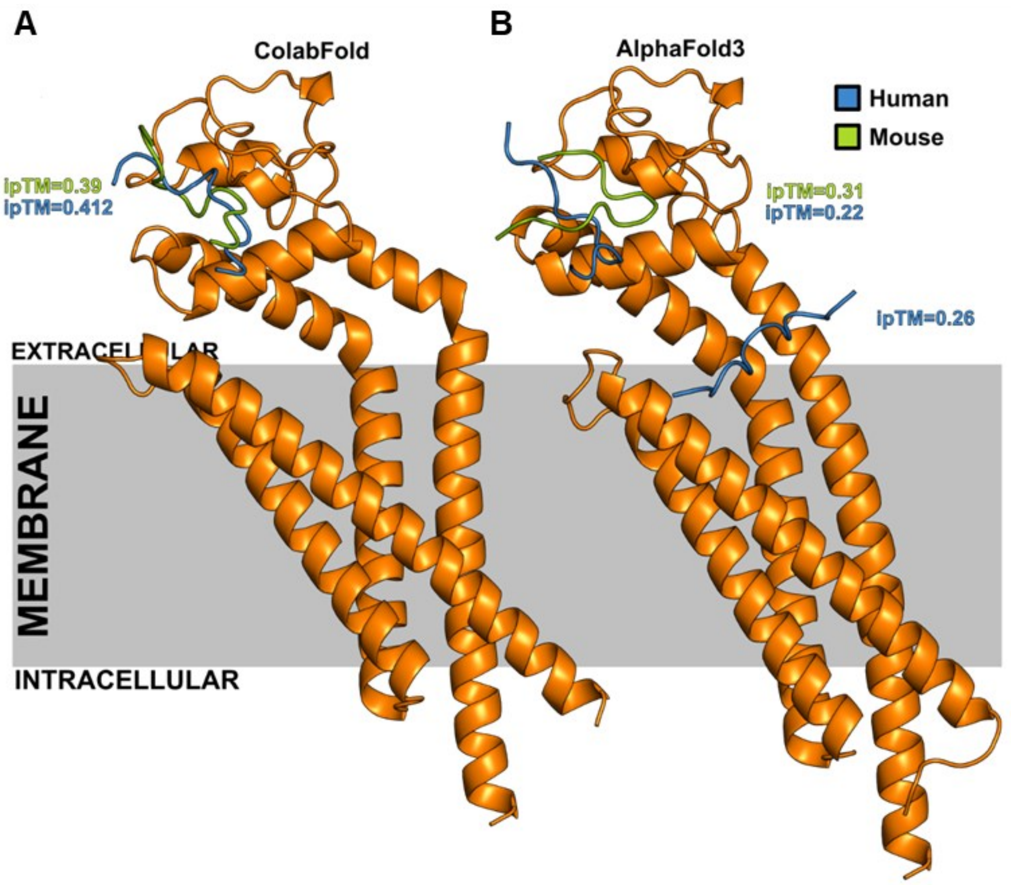

Figure S5. Human and mouse CD63-EP9-glycine spacer complexes as modeled with ColabFold and AlphaFold3.

Models of the complex formed by human or mouse CD63 with EP9 attached to the glycine spacer (GGGK(FAM)C) (see Figure 1) were generated as in Figure 6 with ColabFold and AlphaFold 3. The terminal cysteine was changed to a methionine to avoid spurious formation of disulfide bridges. **A:** ColabFold models show comparable interface predicted TM scores (ipTM) and binding of EP9-glycine spacer in the same EC2 groove as when modeled without the glycine spacer (see Figure 6). **B:** AlphaFold 3 models show overall lower scores, with the top-ranked human CD63 model using the same cavity as without the spacer, and lower-ranked models using the EC2 groove. Mouse complexes use only the groove in EC2 as in ColabFold models.

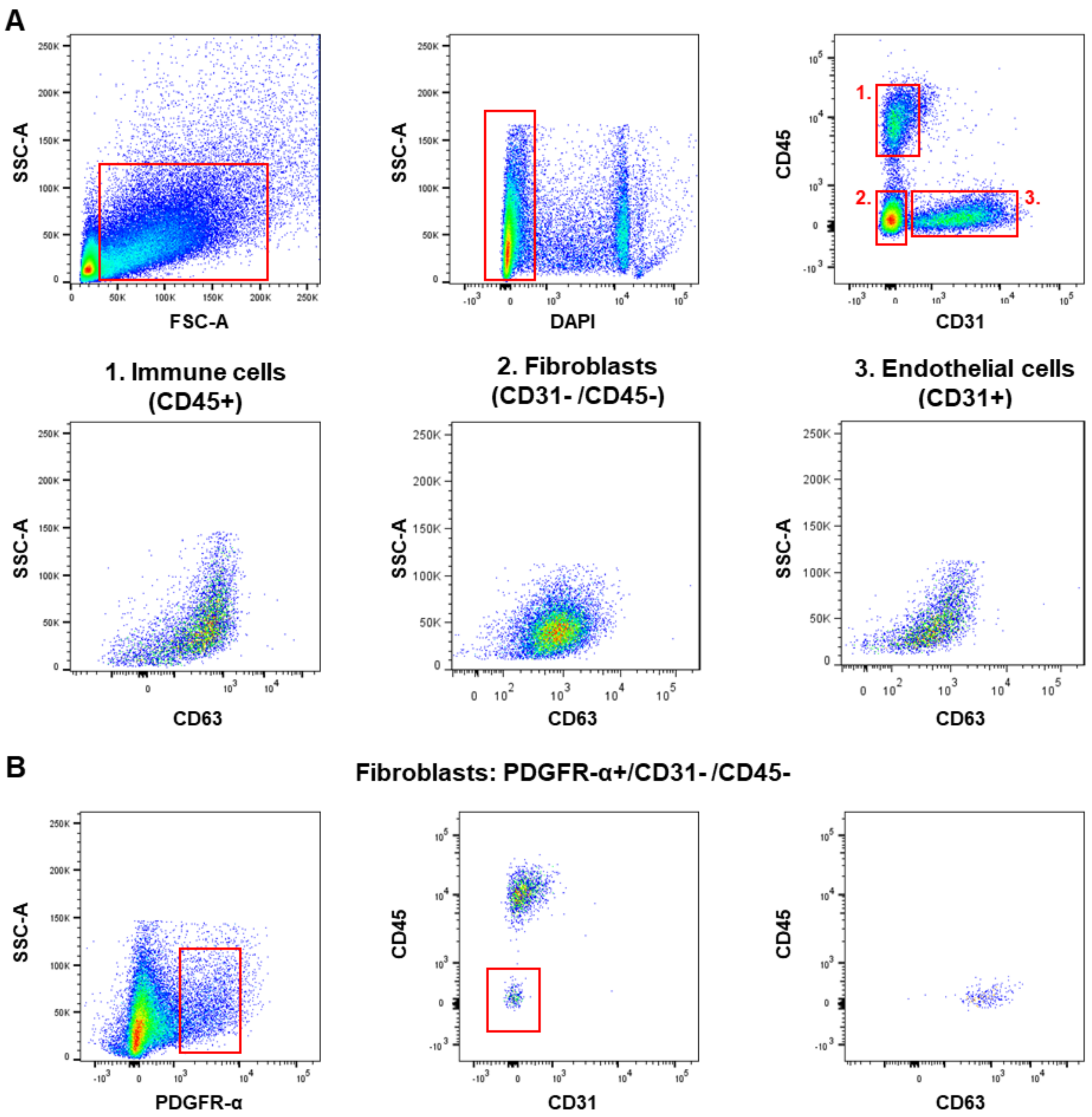

Figure S6. Gating strategy of the flow cytometric analysis of CD63 surface expression in cells isolated from mouse hearts.

Gating strategy applied in the flow cytometric analysis shown in Figure 7. **A:** Gating for living (DAPI-) cardiac fibroblasts (CD31-/CD45-), endothelial cells (CD31+), and immune cells (CD45+). **B:** Gating for PDGFR-α+/CD31-/CD45- cardiac fibroblasts.

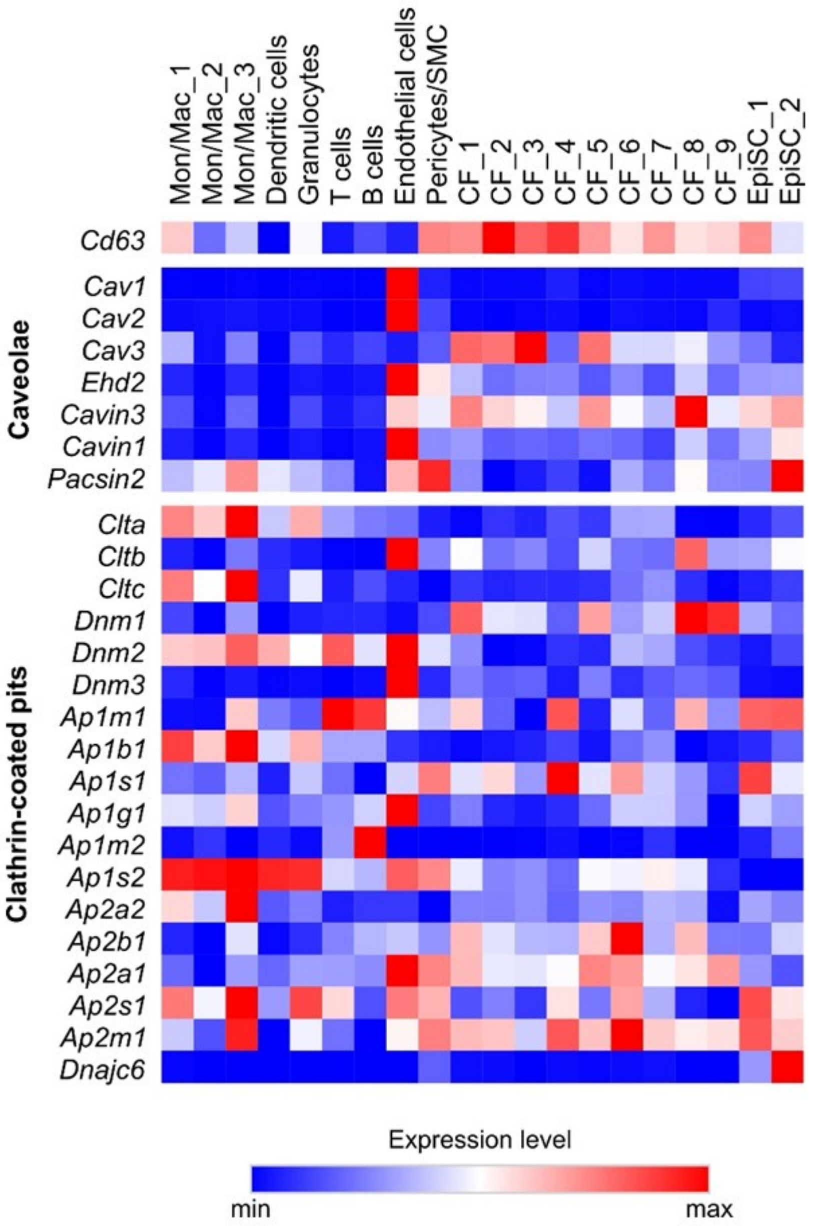

Figure S7. Gene expression of proteins involved in endocytosis pathways by caveolae and clathrin-coated pits in cardiac cell types 5 days post-MI.

Single cell-RNA sequencing (scRNAseq) data from mouse hearts at 5 days post-MI (50 min ischemia/reperfusion) published previously [30,31] were re-analyzed for gene expression of proteins associated with endocytosis pathways. Heat map showing gene expression levels of caveolae proteins [49] and clathrin-coated pit proteins among the identified cardiac cell type populations.

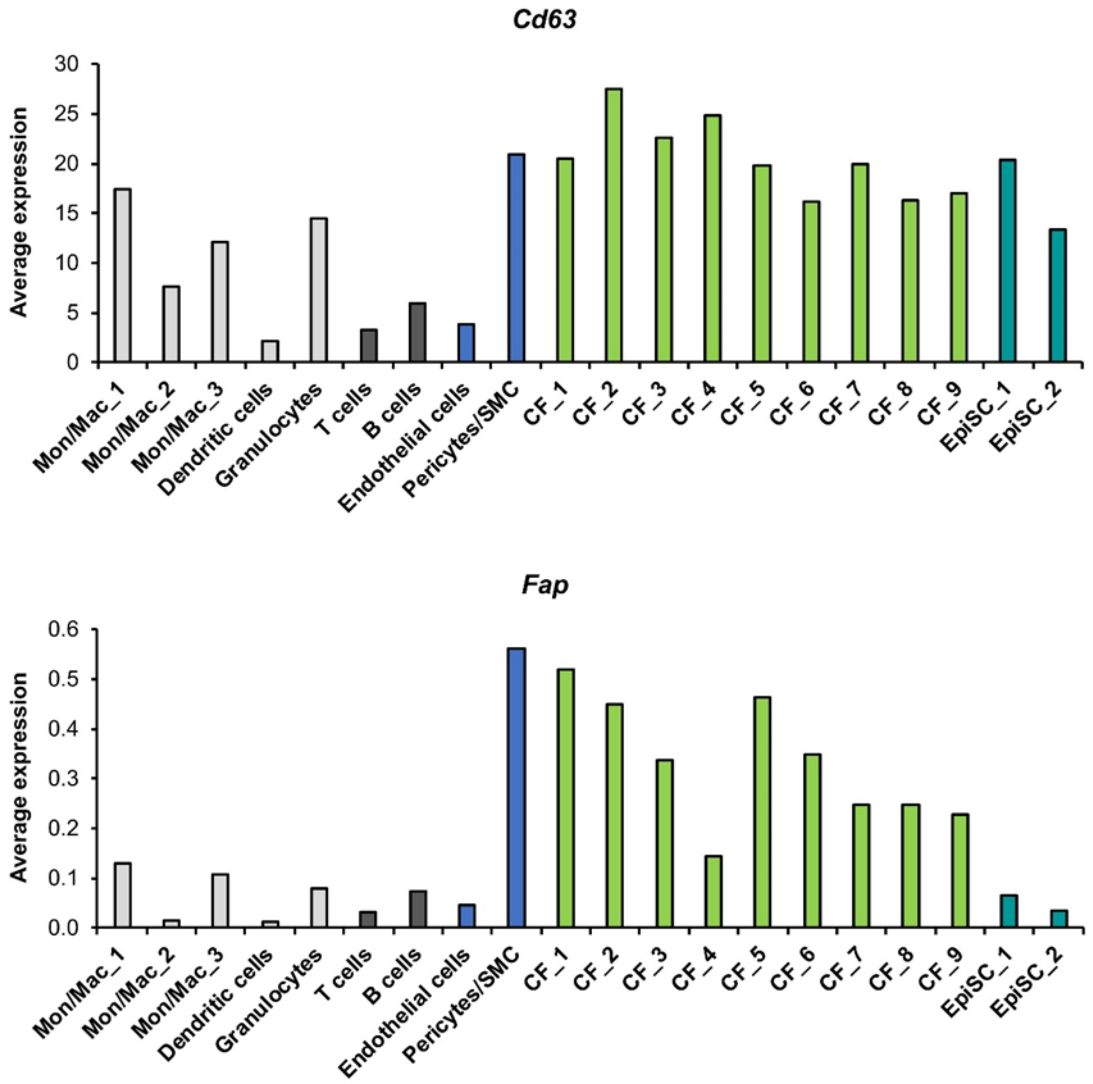

Figure S8. CD63 and FAP gene expression in cardiac cell types 5 days post-MI.

Single cell-RNA sequencing (scRNAseq) data from mouse hearts (n = 3) at 5 days post-MI (50 min ischemia/reperfusion) published previously [30,31] were re-analysed for average gene expression levels of CD63 and FAP in the identified cardiac cell type populations.

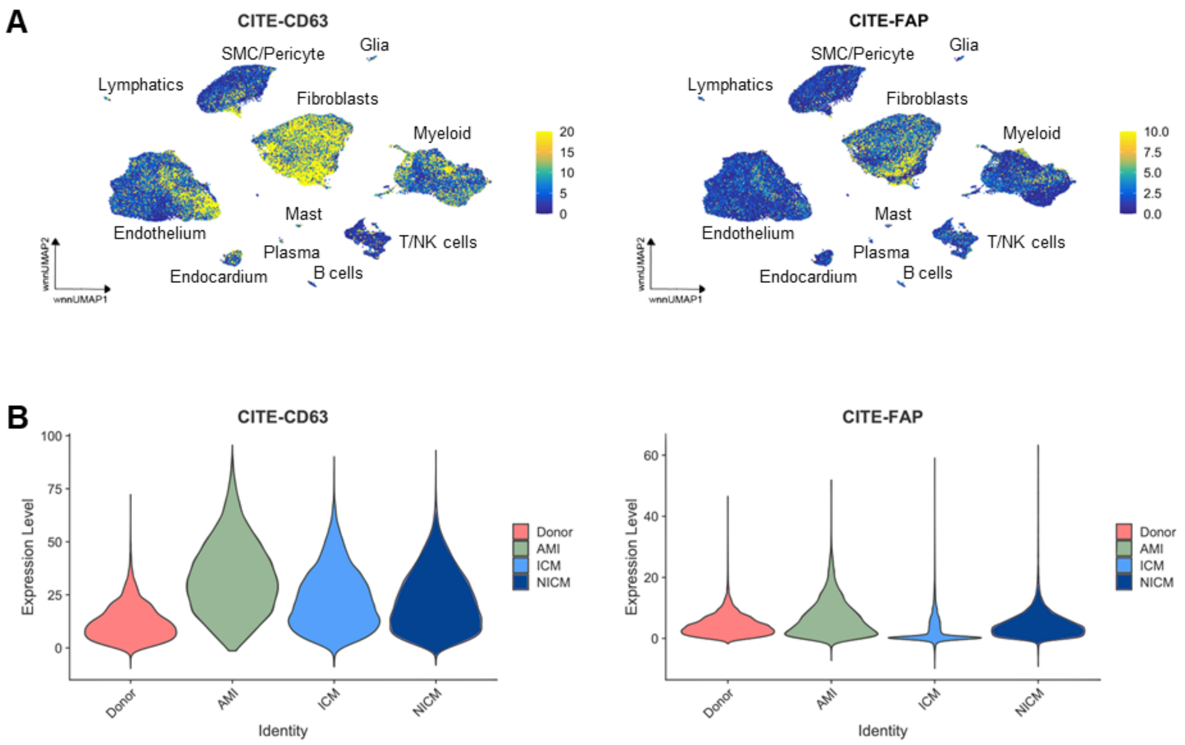

Figure S9. CD63 and FAP protein expression in the healthy and failing human heart.

The previously published integrated CITE-seq (cellular indexing of transcriptomes and epitopes by sequencing) data set of samples from healthy and failing human hearts from 22 individuals [32] was re-analysed for CD63 and FAP protein expression. **A:** Feature plots visualizing CITE-seq protein expression levels of CD63 and FAP among the identified cardiac cell types. **B:** Violin plots generated from the integrated data set visualizing CD63 and FAP protein expression levels in the cardiac fibroblast cell fraction, dissected for the samples from healthy donors (Donor, n = 6), acute MI patients (AMI, < 3 months post MI; n = 4), ischemic cardiomyopathy patients (ICM, > 3 months post-MI; n = 6), and non-ischemic cardiomyopathy patients (NICM, idiopathic dilated cardiomyopathy; n = 6).

Table S1: Amino acid sequences of peptides used in this study.

Sequences of EP peptides targeting EpiSC as recently identified by us [17] as well as sequences of scrambled and mutated (MUT) peptides derived from EP9. For the scrambled peptide, the amino acid positions of EP9 were changed and lysine was replaced by glycine. For the MUT peptide, the scrambled peptide sequence was modified by exchanging prolines for glycine and alanine.

Peptide	Amino acid sequence
**EP1**	SEPIVPL
**EP2**	ATKTIAP
**EP3**	THVYRDE
**EP7**	QSHALMA
**EP9**	KLMLPRP
**Scrambled**	PMGPLLR
**MUT**	GMGALLR

Table S2: Transmembrane proteins identified by ligand-receptor capture (LRC).

List of the 35 transmembrane proteins identified in the ligand-receptor capture (LRC) experiments with the EP9 peptide in comparison to the mutated (MUT) peptide (n = 4 samples each, see Figure 5) on human dermal fibroblasts (NHDF). Protein abundance levels were determined by intensity-based absolute quantitation (iBAQ).

Protein names	Gene names	-Log Welch's t-test p-value iBAQ EP9_iBAQ MUT	Welch's t-test difference iBAQ EP9_iBAQ MUT
**Ryanodine receptor 3**	*RYR3*	0.708926	0.597881
**Tubulin beta-3 chain**	*TUBB3*	0.568849	0.44557
**CD276 antigen**	*CD276*	0.718827	-2.70493
**Sterol O-acyltransferase 1**	*SOAT1*	0.103859	0.268179
**Protein transport protein Sec61 subunit alpha isoform 1; Protein transport protein Sec61 subunit alpha isoform 2**	*SEC61A1; SEC61A2*	0.130541	0.663757
**Receptor expression-enhancing protein;Receptor expression-enhancing protein 5**	*REEP5*	0.0248244	-0.189057
**Tetraspanin; CD63 antigen**	*CD63*	1.55082	4.37498
**Adipocyte plasma membrane-associated protein**	*APMAP*	0.722739	0.991301
**CD44 antigen**	*CD44*	0.188121	-0.794864
**Peptidyl-tRNA hydrolase 2, mitochondrial**	*PTRH2*	1.22198	-3.21273
**Transmembrane and coiled-coil domain-containing protein 1**	*TMCO1*	0.301284	1.47
**Very-long-chain enoyl-CoA reductase**	*TECR*	0.112734	-0.550641
**Membrane-associated progesterone receptor component 1**	*PGRMC1*	0.329259	1.87881
**Chloride intracellular channel protein 1**	*CLIC1*	0.377834	0.550031
**Surfeit locus protein 4**	*SURF4*	0.120849	0.83785
**Monocarboxylate transporter 4**	*SLC16A3*	0.839619	-3.59458
**Monocarboxylate transporter 2**	*SLC16A7*	0.416838	-1.51615
**Vesicle-trafficking protein SEC22b**	*SEC22B*	0.0854926	-0.23306
**Mitochondrial import receptor subunit TOM70**	*TOMM70 A*	0.210034	0.278761
**Transferrin receptor protein 1; Transferrin receptor protein 1, serum form**	*TFRC*	0.284364	0.531218
**Dolichyl-diphosphooligosaccharide--protein glycosyltransferase subunit 1**	*RPN1*	0.337286	1.11898
**ADP/ATP translocase 2;ADP/ATP translocase 2, N-terminally processed**	*SLC25A5*	0.10499	0.521529

**ADP/ATP translocase 3; ADP/ATP translocase 3, N-terminally processed; ADP/ATP translocase 1**	*SLC25A6; SLC25A4*	0.0869138	0.162624
**Voltage-dependent anion-selective channel protein 1**	*VDAC1*	0.0396445	-0.0951638
**Plasma membrane calcium-transporting ATPase 4**	*ATP2B4*	0.0480805	-0.0875998
**Dipeptidyl peptidase 4; Dipeptidyl peptidase 4 membrane form; Dipeptidyl peptidase 4 soluble form**	*DPP4*	0.143485	0.375597
**Transmembrane emp24 domain-containing protein 10**	*TMED10*	0.305242	1.66475
**Matrix metalloproteinase-14**	*MMP14*	0.825034	-2.39069
**Protein transport protein Sec61 subunit beta**	*SEC61B*	0.386119	-2.40084
**Dermcidin; Survival-promoting peptide;DCD-1**	*DCD*	0.14632	-1.21302
**Cytoskeleton-associated protein 4**	*CKAP4*	0.783531	1.37821
**Leucine-rich repeat-containing protein 59**	*LRRC59*	0.243707	0.545554
**Vesicle-associated membrane protein-associated protein A**	*VAPA*	0.556227	1.13898
**Ribosome-binding protein 1**	*RRBP1*	0.16842	-0.553312
**Chloride intracellular channel protein 4**	*CLIC4*	0.316209	1.30194
